# Potato Virus Y Infection Alters Small RNA Metabolism and Immune Response in Tomato

**DOI:** 10.3390/v11121100

**Published:** 2019-11-27

**Authors:** Maria I. Prigigallo, Maja Križnik, Domenico De Paola, Domenico Catalano, Kristina Gruden, Mariella M. Finetti-Sialer, Fabrizio Cillo

**Affiliations:** 1 Consiglio Nazionale delle Ricerche, Istituto per la Protezione Sostenibile delle Piante, G. Via Amendola 122/D, 70126 Bari, Italy; mariaisabella.prigigallo@ipsp.cnr.it; 2 National Institute of Biology, Department of Biotechnology and Systems Biology, Večna pot 111, 1000 Ljubljana, Slovenia; maja.kriznik@nib.si (M.K.); kristina.gruden@nib.si (K.G.); 3 Consiglio Nazionale delle Ricerche, Istituto di Bioscienze e BioRisorse, Via G. Amendola 165/A, 70126 Bari, Italy; domenico.depaola@ibbr.cnr.it; 4 Consiglio Nazionale delle Ricerche, Istituto di Tecnologie Biomediche, Via G. Amendola 122/D, 70126 Bari, Italy; domenico.catalano@cnr.it

**Keywords:** RNA silencing, plant defense response, *Solanum lycopersicum*, *Potato virus Y*, molecular plant-virus interactions, microRNA, secondary small interfering RNA, phasiRNA, small RNA sequencing

## Abstract

*Potato virus Y* (PVY) isolate PVY^C^-to induces growth reduction and foliar symptoms in tomato, but new vegetation displays symptom recovery at a later stage. In order to investigate the role of micro(mi)RNA and secondary small(s)RNA-regulated mechanisms in tomato defenses against PVY, we performed sRNA sequencing from healthy and PVY^C^-to infected tomato plants at 21 and 30 days post-inoculation (dpi). A total of 792 miRNA sequences were obtained, among which were 123 canonical miRNA sequences, many isomiR variants, and 30 novel miRNAs. MiRNAs were mostly overexpressed in infected vs. healthy plants, whereas only a few miRNAs were underexpressed. Increased accumulation of isomiRs was correlated with viral infection. Among miRNA targets, enriched functional categories included resistance (*R*) gene families, transcription and hormone factors, and RNA silencing genes. Several 22-nt miRNAs were shown to target *R* genes and trigger the production of 21-nt phased sRNAs (phasiRNAs). Next, 500 phasiRNA-generating loci were identified, and were shown to be mostly active in PVY-infected tissues and at 21 dpi. These data demonstrate that sRNA-regulated host responses, encompassing miRNA alteration, diversification within miRNA families, and phasiRNA accumulation, regulate *R* and disease-responsive genes. The dynamic regulation of miRNAs and secondary sRNAs over time suggests a functional role of sRNA-mediated defenses in the recovery phenotype.

## 1. Introduction

In the last two decades, thanks to the recognition of noncoding small RNAs (sRNAs) as leading regulatory factors, studies on long-established mechanisms that intervene in the evolution, development, and control of many biological processes have seen an impressive degree of progress. Different classes of sRNAs have been identified to date as important regulators of gene expression and adaptive responses to adverse conditions [1,2]. sRNAs regulate different plant biological processes, such as physiology, growth, development, chromatin remodeling, and the proliferation of transposable elements [2,3]. More recently, a key role of sRNA-guided processes has been proposed in defense responses, and investigation of their involvement in different plant–pathogen interaction mechanisms is still a topic of intense investigation. Notably, micro (mi)RNAs and other sRNA species that modulate immune responses by targeting disease resistance genes have been the focus of many studies [4,5,6,7,8].

The classification of sRNAs relies on the origin and biogenesis of these molecules. Plant sRNAs can be broadly classified into miRNAs and small interfering RNAs (siRNAs), first described as part of a complex, RNA-based antiviral protection system. The mechanism that produces sRNAs entails the cleavage of double-stranded RNA (dsRNA), or of single-stranded RNAs forming stable secondary structures of endogenous or exogenous origins, yielding miRNAs or siRNAs of predominantly 21 to 24 nucleotides (nt) in length. Precursors of sRNAs are processed by Dicer-like (DCL) enzymes and then loaded into Argonaute (AGO) proteins to form the RNA-silencing complexes (RISC). RISC is guided to complementary target mRNA sites to direct cleavage or translational repression depending on the extent of complementarity [9]. In tomato (*Solanum lycopersicum* L.), 28 RNA silencing-associated genes have been identified, including seven DCLs, fifteen AGOs, and six RNA-dependent RNA polymerases (RDRs). The expression profiles of many of these genes were shown to be altered by viral infections [10]. 

However, differing from miRNAs, siRNAs may undergo a further enrichment process based on the activity of host RDRs [11,12]. In addition, different classes of siRNAs involved in plant gene regulation and defense mechanisms are emerging, such as the heterochromatic siRNAs (hc-siRNAs), natural antisense transcript siRNAs (nat-siRNAs), and phased secondary siRNAs (phasiRNAs). This latter category also includes the well-characterized, trans-acting siRNAs (ta-siRNAs), originating from *TAS* transcripts, that silence their targets in trans [13,14]. phasiRNAs originate from phasiRNA-generating genomic regions (*PHAS* loci), and their biogenesis is triggered by miRNAs targeting *PHAS* loci transcripts. phasiRNAs play an important role in disease resistance, through the targeting of gene families encoding large groups of nucleotide-binding site (NBS)–leucine-rich repeat (LRR) receptor proteins (NLRs or R proteins) and several receptor-like proteins (RLPs) and receptor-like kinases (RLKs), also harboring LRR domains [6,12].

Viral infection triggers RNA silencing (RS), an antiviral plant response which serves to degrade viral genomes. Viral RNA genomes present self-paired sequences that form secondary structures and replicate via dsRNA intermediates. Viral dsRNAs are recognized by DCLs and cleaved into 21 to 24-nt viral(v) siRNAs, giving rise to RS-based antiviral immune responses [15]. Similar to plant sRNAs, vsiRNAs are recruited into different AGO proteins forming RISC to induce the degradation of additional viral RNA molecules entering the plant cell [16]. As a counter defense, viruses have evolved to encode proteins that operate as suppressors of RNA silencing (VSRs), hindering the plant antiviral response [17,18]. VSRs belong to a heterogeneous group of proteins that suppress RS mechanisms at multiple steps. Symptoms induced during viral infections have often been explained by the interference of VSRs with the developmental pathways controlled by miRNAs or other sRNA-regulated cellular processes [19,20,21,22]. 

Plants infected by viruses can undergo symptom recovery, usually leading to symptom remission coupled with a decrease of viral accumulation levels. This phenomenon has been described in diverse plant–virus associations where, despite the efficient viral entry and the initial host invasion, a decrease of disease phenotype was manifested in later stages of the infection. The recovery phenotype has been associated with RS mechanisms that lead to the suppression of viral RNA accumulation [23,24].

*Potato virus Y* (PVY), the type species of genus *Potyvirus* in the family *Potyviridae*, is a pathogen of agricultural importance, ranked as the fifth most important plant virus worldwide, according to a survey within the plant virology community [25]. PVY infects several crop species in the plant family *Solanaceae*, and represents a significant threat to potato, pepper, and tomato productions. Its rapid nucleotide mutation and genome recombination rates may contribute to the emergence of novel strains, changing the virulence of the virus, usually towards increased aggressiveness, and consequently, to worse symptomatology of the plant [26,27]. It has been shown that PVY infection selectively alters the expression of sRNAs in its solanaceous hosts depending on the plant sensitivity. Certain sRNAs have been shown to be involved in the establishment of tolerance and resistance responses to PVY infection in potato and tobacco plants [13,28]. Additionally, several studies have reported that, similar to other plant hosts, tomato responds to viral pathogens by generating infection-responsive sRNAs of different classes including miRNAs and phasiRNAs [29,30,31,32,33]. In this study, we examined PVY-tomato interactions, looking into the complexity and diversity of defense pathways involving sRNAs. We used a combination of experimental and computational approaches in order to identify and characterize PVY-responsive sRNAs in infected tomato plants. We generated four sRNA libraries from PVY^C^-to-infected and mock-inoculated tomato leaves at two time points in order to capture the process of recovery in tomato. Using high-throughput sequencing (sRNA-seq), we performed a genome-wide identification and expression profiling of miRNAs and secondary sRNAs. The data herein offer new insights into sRNA-regulated host responses to PVY infection, and indicate a functional role of sRNAs in the molecular mechanisms through which disease tolerance in tomato is controlled.

## 2. Materials and Methods 

### 2.1. Plant and Virus Material

A PVY isolate belonging to the strain PVY^C^, found on cultivated tomato in Italy and named PVY^C^-to (accession no. EU482153), was maintained on and purified from tobacco (*Nicotiana tabacum* cv. Samsun) plants as described [34]. Two-week-old seedlings of tomato cv. UC82 were used for plant inoculation. For PVY inoculation, a 10 μL aliquot of a purified virus preparation at 50 ng/μL in 30 mM Na_2_HPO_4_, or the same buffer alone as the negative mock-inoculated control, was rubbed on the cotyledons of twelve plants per inoculum. 

### 2.2. RNA Extraction

Symptomatic and recovered (symptom-free) leaves from six PVY^C^-to-inoculated plants were collected at 21 and 30 dpi, respectively. Leaves from six mock-inoculated plants were sampled at the same time points as the negative controls. The leaves from each treatment were pooled together into four samples. 

Total RNA was extracted from about 100 mg of leaf tissue using the TRIzol reagent (Thermo Fisher Scientific, Waltham, MA USA) according to the manufacturer’s instructions. RNA preparations were subjected to DNase digestion using TURBO DNA-*free*™ Kit (Thermo Fisher Scientific) and resuspended in nuclease-free water. Total RNA was used for the detection of viral RNA and sRNA sequencing. The leaves from an additional set of six mock- and six PVY^C^-to-inoculated tomato plants at 21 dpi were collected and pooled into three biological replicates per treatment for separate extraction of high and low molecular weight RNA fractions (HMW and LMW, respectively) using the mirVana miRNA isolation kit™ (Ambion, Austin TX, USA), according to the manufacturer’s instructions. These RNA preparations, after DNase treatment, were used for gene and miRNA quantification by reverse transcription-quantitative polymerase chain reaction (RT-qPCR). RNA integrity was estimated by agarose gel electrophoresis on a 1.2% agarose gel. RNA concentration and purity were measured with a Nanodrop ND-1000 Spectrophotometer (Nanodrop Technologies, Thermo Fisher Scientific).

### 2.3. Primer Design and RT-qPCR Analysis for Detection of Viral RNA, mRNAs and miRNAs

Six RS-related and four disease-related genes were selected among those described as miRNA targets in tomato. Primer pairs used for the tomato gene quantification or PVY genomic RNA detection were designed with the support of the IDT’s PrimerQuest software (http://eu.idtdna.com/Scitools/Applications/Primerquest/) (Table S11). For viral RNA and tomato mRNA quantitative analysis, RT-qPCR was performed as previously described [35]. Briefly, RNA samples were reverse-transcribed using the High Capacity cDNA Reverse Transcription Reagents (Thermo Fisher Scientific), according to the manufacturer’s instructions. qPCR reactions were performed in a 96-well CFX96 RealTime PCR System (Bio-Rad, Hercules, CA, USA) using 2X PowerUp™ SYBR Green PCR Master Mix (Thermo Fisher Scientific), 400 nM forward and reverse primers, and 1 μL of a 1:10 dilution (approx. 5 ng) of reverse-transcribed RNA in a total volume of 10 μL. 

The miRNA 1st-Strand cDNA Synthesis Kit and miRNA QPCR Master Mix (Agilent Technologies) were used for RT-qPCR analysis of 21 miRNAs in order to validate sRNA-seq experiments. cDNA was amplified using the provided universal reverse primer and the miRNA-specific forward primers (Table S11) according to the manufacturer’s instructions. Forward primers were homologous to the selected known and novel miRNAs, and included an additional 6-nt sequence at the 5’ terminus for the amplicon stabilization [22].

Relative viral RNA, mRNA, and miRNA expression levels were calculated according to the (2^–ΔΔCt^) method using tomato *UBI3* (for viral RNA, mRNAs) or both tomato snoU6 and miR167a (for miRNAs) as the endogenous controls and corrected for PCR amplification efficiencies as previously described [22,35]. Data were statistically analyzed by Student’s t-test.

For the validation of sRNA-seq data with relative miRNA expression levels estimated by RT-qPCR, sRNA-seq relative expression values were calculated by summing the normalized RPM counts of mature miRNAs that perfectly matched with the specific forward primers used for RT-qPCR, and RPM counts of all isomiRs that matched at least three nucleotides at the 3’-end of the primers.

### 2.4. Sequencing and sRNA Data Analysis

Four high quality sRNA libraries were constructed using the TruSeq Small RNA Library Prep Kit (Illumina) and sequenced in a single-read mode (50 bp) on a HiSeq 2000 (Illumina) by IGA Technology Services (Udine, Italy).

The raw sRNA sequencing reads were first trimmed to remove adaptor sequences using the cutadapt tool (https://cutadapt.readthedocs.io/en/stable/guide.html) [36] and further quality-assessed using FastQC (https://www.bioinformatics.babraham.ac.uk/projects/fastqc). The raw reads were filtered using the Filter Tool of the UEA sRNA Toolkit [37] by discarding low complexity reads (containing at most two distinct nucleotides), reads shorter than 18 nt and longer than 26 nt, as well as reads matching rRNAs, tRNAs, snRNAs, and snoRNAs in the RNACentral database (https://rnacentral.org) [38]. 

To identify known tomato miRNAs, filtered sRNA reads were compared to tomato miRNAs registered in the miRBase database release 22 (http://www.mirbase.org) [39], allowing no mismatches. 

To identify novel unannotated miRNAs and their loci of origin (*MIR* loci), reads were submitted to the two plant miRNA prediction tools ShortStack 3.8.3 [40] and miR-PREFeR [41]. Predictions were performed using default parameters, except that no mismatches were allowed during mapping on the reference tomato genome (Genome build SL3.0, solgenomics.net). As novel miRNAs were considered only with more than ten raw reads in at least two sRNA libraries, the miRNA sequence and corresponding miRNA* and *MIR* locus should have been predicted with both miRNA prediction tools. Within the prediction analyses, the reads that could be mapped to more than 30 locations in the tomato genome were also discarded as being too repetitive to be miRNAs. The output of miRNA prediction tools also contained the predictions of already annotated tomato *MIR* loci; therefore, to separate them from potential novel *MIR loci* candidates, annotated pre-miRNA precursors from the miRBase database release 22 were mapped to the reference tomato genome (Genome build SL3.0, solgenomics.net) using bowtie2 (http://bowtie-bio.sourceforge.net/bowtie2/index.shtml) [42]. Next, genome locations were extracted and compared with predicted *MIR* loci locations using our internally developed script. If no overlap was detected, the predicted *MIR* loci were regarded as novel. Novel tomato miRNAs were further classified into known or novel miRNA families by clustering their predicted pre-miRNA sequences with sequences of known plant pre-miRNAs from miRBase using CD-HIT-EST with an identity threshold of 0.8 (http://weizhong-lab.ucsd.edu/cdhit-web-server/cgi-bin/index.cgi?cmd=cd-hit-est) [43]. The sequences showing similarities with annotated pre-miRNAs were grouped into corresponding known miRNA families, and sequences that did not show similarity with known plant miRNAs were classified as novel.

Additionally, sequence miRNA variants (isomiRs) of known and novel miRNAs were identified using computational pipeline isomiRID [44]. Only sRNAs perfectly matching known or novel pre-miRNA sequences, known as templated isomiRs [45], were considered. In addition, to ensure robustness of isomiR predictions, only sequences present at a minimum of 5 raw reads in at least two sRNA libraries were regarded as isomiRs.

Predictions of *PHAS* loci was performed using ShortStack 3.8.3. *PHAS* loci were detected by mapping preprocessed sRNA reads to tomato transcriptome sequences (ITAG release 3.2, solgenomics.net). Analyses of phasing were performed in 21- and 24-nt intervals using the default settings.

To identify viral (v)siRNAs, reads of lengths 20–24 nt from all PVY^C^-to-infected samples were mapped to the PVY^C^-to isolate-specific genome (accession no. EU482153) using CLC Genomics Workbench version 8 (http://www.clcbio.com/), allowing 100% identity.

### 2.5. Identification of Differentially Expressed miRNAs

Preprocessed reads from sRNA-seq samples were mapped with no mismatches to all identified known, novel miRNAs and isomiRs, and counted. sRNA counts were further normalized to total library sizes. To identify PVY-responsive miRNAs, normalized miRNA counts (given in RPM) were compared between PVY and mock samples. miRNAs accumulating beyond a threshold of ±2 log_2_ fold change (log_2_fc) in infected vs. healthy plants and at least 1 RPM (approx. 20 raw reads in one of the data set) were considered differentially expressed. miRNA expression profiles were subjected to hierarchical clustering data analysis using the online ClustVis tool (https://biit.cs.ut.ee/clustvis/) [46] and applying the following parameters to the analysis: Clustering distance for rows-Euclidean; Clustering method for rows: complete; Tree ordering for rows: tightest cluster first; and Number of clusters in rows: 30. A heat map was generated to show the changes of each miRNA expression according to time course after PVY^C^-to infection, and miRNAs showing similar expression pattern were grouped.

### 2.6. Target Gene Prediction and Functional Analysis

In silico identification of tomato transcripts targeted by differentially expressed miRNAs (DEMs) was carried out using the psRNATarget v2 (http://plantgrn.noble.org/psRNATarget/) [47] and tomato transcriptome sequences (ITAG release 3.2, solgenomics.net) using default parameters, except that a stricter maximum expectation parameter was applied (Expectation: 3.0).

Results of miRNA-target (*PHAS* loci) interactions were used to reveal miRNA triggers of the phasiRNA production. Only 22-nt miRNAs were kept as potential triggers [48,49].

A phylogenetic analysis was performed for the 19 disease-related *NLRs* and *RLP/RLKs* genes, selected by generating phasiRNAs and representing targets of 22-nt miRNAs. Only genes with a phasescore higher than two were included in the analysis. Selected *NLR/RLK/RLP* genes and additional tomato genes active in resistance against fungi, nematodes, and viruses, retrieved from the literature (Table S12) were used to infer a phylogenetic tree. All sequences were aligned using ClustalW within the BioEdit program (http://www.mbio.ncsu.edu/BioEdit/bioedit.html) [50], considering only the C-terminal LRR domain superfamily. The neighbor-joining method [51] was used to build up the phylogenetic tree using the Molecular Evolutionary Genetics Analysis (MEGA) software version X (https://www.megasoftware.net/home) [52] with a bootstrap of 1000 replicates. The tomato *Ubiquitin* gene sequence (Solyc11g012950) was used as the out-group.

All DEMs were analyzed for functional over-representation in biological pathways with the MapMan software (https://mapman.gabipd.org/) [53]. miRNAs were grouped into MapMan s according to their predicted targets determined in silico and by Degradome-Seq analysis, and using tomato ontology information (for version ITAG3.2) (http://www.gomapman.org/export/current/mapman). Moreover, a Wilcoxon rank sum test was carried out to identify the pathways in which miRNAs underwent coordinated changes [53].

### 2.7. Degradome-Seq Target Validation

Six degradome datasets produced from virus-infected and healthy tomato leaves [30,54] were analyzed with CleaveLand4 (http://sites.psu.edu/axtell/software/cleaveland4/) [55] using all our experimentally-identified miRNAs and the tomato transcriptome sequences (ITAG release 3.2). All identified degradation targets were classified into five categories (0 to IV) as previously described [56]. Only categories with high confidence of cleavage (0, I, II, III) were considered for biological interpretation. The results of miRNA-target (*PHAS* loci) interactions were also used to confirm miRNA triggers of the phasiRNA production determined in silico.

### 2.8. Validation of miRNA Targets with RLM 5´-RACE

An RNA Ligase-Mediated Rapid Amplification of cDNA Ends (RLM 5’-RACE) protocol [57] was used to validate miRNA cleavage events of selected *NLR* genes in PVY-infected tomato leaf tissues. Four gene-specific reverse primer pairs and a common forward primer for semi-nested PCR were designed based on the downstream sequence of the predicted miRNA-guided cleavage site of *NLR* transcripts *Cf-9/Solyc01g005870* and *R1A-4/Solyc05g008070* using the IDT’s PrimerQuest software (Table S11). Four µg of HMW RNA were ligated to a synthetic 5’ HD adapter (Table S11) using a T4 RNA ligase (New England Biolabs, Beverly, MA, USA) as described [58]. After purification with the RNA Clean & Concentrator kit™ (Zymo Research, Orange, CA, USA), and the RT reaction was set up using gene-specific reverse primers 5RACE-2_REV (Table S11) and SuperScript^®^ IV Reverse Transcriptase (Thermo Fisher Scientific). A semi-nested PCR assay was then performed to amplify the cleaved 5′-end using 5’ HD adapter-specific forward primer RP-1_FOR in both amplification rounds, while reverse primers were gene-specific 5RACE-2_REV and 5RACE-1_REV in the first and the second amplification round, respectively (Table S11). The first and second PCR rounds were performed in a final volume of 50 µL containing 1.25 U DreamTaq DNA Polymerase, 1X DreamTaq Green Buffer (Thermo Fisher Scientific), 400 nM of each forward and reverse primer, 200 nM of each dNTP, and 2 µL of cDNA (or 1 µL of 1:100 diluted templates from the first-round PCR). The amplification conditions of the first-round PCR consisted of 60 s at 95 °C, followed by 10 cycles of 95 °C for 30 s, 60 °C for 30 s, and 72 °C for 30 s, followed by a final step of 72 °C for 5 min. For the second-round PCR, conditions were 60 s at 95 °C, followed by 35 cycles of 95 °C for 30 s, 60 °C for 30 s, and 72 °C for 20 s, and a final step of 72 °C for 5 min. All PCRs were conducted in a T100™ Thermal Cycler (Bio-Rad).

Amplicons from the second round PCR were separated by 1.2% agarose gel electrophoresis. Bands of the expected size (approx. 180–200 bp) were excised from the agarose gel, purified with QIAEX II^®^ Gel Extraction Kit (Qiagen, Hilden, Germany) and cloned into chemically-competent *E. coli* DH5α cells using the pGEM®-T Easy Vector (Promega, Madison, WI, USA), following the manufacturer’s protocol. For each cloned amplicon, about 10 clones were selected and sequenced with the M13 reverse primer (Table S12) at Macrogen Europe (Amsterdam, the Netherlands). Sequences were aligned using MultAlin software (http://multalin.toulouse.inra.fr/multalin/) [59] and analyzed to identify specific cleavage sites within homologous groups of sequences. 

### 2.9. Data Availability and Retrieval

The sRNA sequencing data herein produced were deposited in NCBI Sequence Read Archive (SRA) database, under the accession number PRJNA563278.

Six degradome datasets (GSM1213988, GSM1213989, GSM1213990, GSM1213991, GSM1372426, and GSM1372427) produced by Feng and coauthors [30] and Bai and coauthors [54] were retrieved from the NCBI Gene Expression Omnibus database under the accession numbers GSE50085 and GSE56974.

## 3. Results

### 3.1. Tomato plants Exhibit Symptom Recovery at Later Stages of PVY^C^-to Infection

Viral infection was detected at 7 dpi in all PVY^C^-to inoculated plants by dot blot hybridization (not shown). Starting from 10 to 12 dpi, PVY^C^-to induced symptoms of mild mosaic, leaf distortion and plant growth reduction. Symptoms reached the highest severity at 21 dpi, when the first set of leaf tissues for RNA extraction was sampled. After nine days (30 dpi), PVY^C^-to infected plants started showing the signs of disease recovery, and newly-grown leaves showed milder disease symptoms (Figure S1a). Symptom observations were repeated and confirmed in three independent experiments. 

### 3.2. Viral siRNA Accumulation Levels Correlate with Viral RNA Titer

To investigate the correlation between viral RNA titer and production of vsiRNAs in symptomatic leaves (21 dpi) and during the following symptom recovery phase (30 dpi), four sRNA libraries were generated from either healthy (mock-inoculated) or infected (PVY^C^-to-inoculated) tomato leaf tissues from both time points.

Libraries yielded 20 million reads per sample on average, with 21–24-nt sRNA constituting the predominant size classes (Table 1, in bold). In libraries from mock-inoculated plants, 24-nt sRNAs were largely prevalent (56 and 53% in Mk-21 and Mk-30, respectively), followed by 23-nt (15%) and similar numbers of 22 and 21-nt sRNA (about 10 and 12% in Mk-21 and Mk-30, respectively). The size distribution of host sRNAs was altered in libraries from PVY-inoculated plants, where 21-nt sRNA were predominant (56 and 40% in PVY-21 and PVY-30, respectively) and 24-nt sRNA were reduced to 16 and 27% in PVY-21 and PVY-30, respectively. Also, 21-nt vsiRNAs accounted for 38 and 27% of sRNAs in PVY-infected samples from symptomatic (21 dpi) and recovered leaves (30 dpi), respectively (Table 1). By measuring the accumulation levels of PVY RNA, we found that level of vsiRNAs in PVY-infected plants positively correlated with the viral genomic RNA concentration. PVY-infected symptomatic leaves at 21 dpi exhibited higher vsiRNAs levels and higher virus RNA titers, whereas the vsiRNA abundance was lower as viral RNA load decreased by about 30% in recovered leaves at 30 dpi (Figure S1b).

### 3.3. Known and Novel miRNAs and Their isomiR Variants Were Identified in Tomato Leaf Tissues 

A total of 175 unique miRNAs were identified belonging to 64 known (i.e., reported in miRBase) miRNA families. Out of those 175, 123 matched to previously described tomato miRNAs and 52 to miRNAs found in other plant species. Of the latter 52 known miRNAs, 47 were miRNA-related isomiR forms (i.e. sequence variants), while 5 were mapped to novel tomato *MIR* genes (Table S1, Figure S2). Using in silico miRNA prediction, 24 novel *MIR* genes were discovered, coding for 30 novel unannotated canonical miRNAs and 73 previously unannotated novel miRNA variants. Based on the similarity of their predicted precursor sequences with other annotated plant precursor sequences, novel miRNA sequences were assigned to 15 known and 11 novel miRNA families (Tables S1 and S2).

Many studies have shown that isomiRs are functional and can act cooperatively with their canonical miRNAs to target common biological pathways [60]. Hence, we examined the diversity of *MIR* loci in production of isomiRs and their biological relevance in the tomato-PVY interaction. Our libraries contained a total 587 previously unannotated isomiRs (Figure S2). Only 19 *MIR* loci did not generate isomiR variants, while 18 *MIR* loci produced more than 10 isomiRs, and 4 of them, namely *MIR6023*, *MIR7981f*, *MIR9474,* and *MIR10532,* were the most diverse, giving rise to more than 20 different miRNA variants. Among novel *MIR* loci, *MIR11* locus generated the highest number of different sequence variants (33) (Table S1).

**Figure 1:**
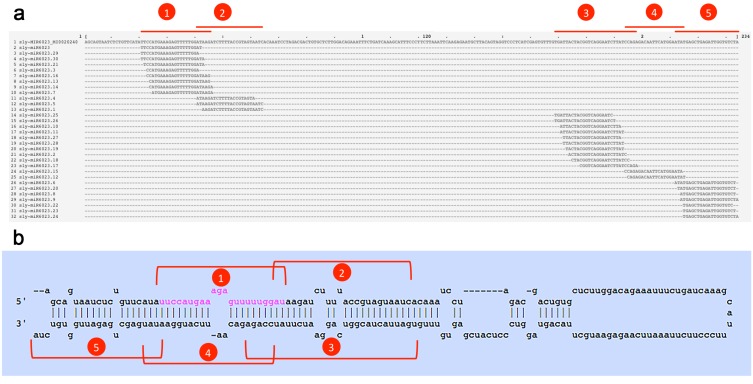
miRNA and isomiR sequences originating from the tomato miR6023 precursor. Five distinct clusters (red brackets 1–5) where the canonical miRNA form, its putative complementary strand (miRNA*) (sly-miR6023.15), and other isomiRs mapped are shown. (**a**) The distribution of 31 unique sRNA reads along the sly-miR6023 precursor (miRBase ID: MI0020240). (**b**) miR6023 precursor fold-back structure from miRBase. In pink, the canonical sequence of sly-miR6023, mapping to cluster 1.

One example of sequence diversity generated from a single *MIR* locus is *MIR6023*, encoding canonical sly-miR6023, a well-characterized miRNA which regulates *R* genes in tomato [4]. Thirty-one sly-miR6023 variants were identified in at least two of the four libraries. By mapping all isomiRs to the pre-miR6023 sequence (miRBase acc. MI0020240), five distinct clusters were identified (Figure 1a). According to the proposed stem-loop structure of the miR6023 precursor, two cluster pairs (Figure 1b, clusters 1–4 and 2–3, respectively) were apparently organized as matching sequences (miRNA/miRNA* duplexes), whereas for cluster 5, a putative miRNA* sequence was not detected (Figure 1b). Five miRNA sequences originating from the miR6023 precursor, one per each isomiR cluster, were quantitatively predominant: the canonical 22-nt sly-miR6023 on the 5p-arm (cluster 1) and its putative miRNA* sequence sly-miR6023.15 (cluster 4); the 22-nt sly-miR6023.28 on the 3p-arm (cluster 3) and its putative miRNA* sequence sly-miR6023.1 (cluster 2); and the 19-nt sly-miR6023.23 on the 3’-terminal region of the 3p-arm (cluster 5) (Table 2). 

### 3.4. Regulation of Known and Novel Tomato mRNA upon PVY^C^-to Infection

We identified 247 and 219 miRNAs at 21 and 30 dpi respectively that were upregulated by PVY infection. Among those, 192 were upregulated at both analyzed time points during infection. On the other hand, 66 and 11 miRNAs at 21 and 30 dpi respectively were downregulated, with only two miRNAs showing downregulation at both time points (Figure S3).

As reported in Table 3, 28 out of 192 miRNAs upregulated at both time points were canonical tomato miRNAs already recorded in miRBase, and 13 were canonical, newly-discovered miRNAs from either known or novel miRNA families. Among downregulated canonical miRNAs, seven were downregulated at 21 dpi, whereas their levels remained unchanged at 30 dpi (Table 3). Interestingly, all novel miRNAs retrieved in the present work were regulated in at least one time point: novel-sly-miR4 was downregulated at 21 dpi, novel-sly-miR10 was upregulated at 30 dpi, and all other mature novel miRNAs were upregulated at both time points (Table 3, Table S1). Many isomiRs also showed altered accumulation levels upon PVY infection; among miRNAs altered at 21 dpi, for instance, 81.4% and 89.4% upregulated and downregulated miRNAs, respectively, were isomiRs (Table S1). In some cases, e.g. sly-miR6022, sly-miR6025 and sly-miR10534, the canonical miRNA forms were less abundant than some of their isomiRs in our libraries (Table S1). 

Some miRNAs were found to accumulate specifically in virus-infected samples whilst being expressed at null or very low levels in healthy plants. Table S3 lists miRNAs that were found in infected libraries (at both time points) while being not or barely detectable in healthy tomato samples. Fifty-three miRNA sequences belonging to 22 known miRNA families were detected only in PVY-infected libraries, and four miRNAs from novel miRNA families also responded to these criteria. A relevant presence of defense-related miRNA families (e.g. sly-miR396, miR398, miR482, miR6022, miR6023, miR6027) was found in this dataset. In the case of sly-miR6023, significantly, 17 out of 31 sly-miR6023 isomiR forms were specifically associated with PVY infection, indicating that the wide diversification of this miRNA family is, at least in part, a direct consequence of viral infection (Table 2, Table S3).

**Figure 2:**
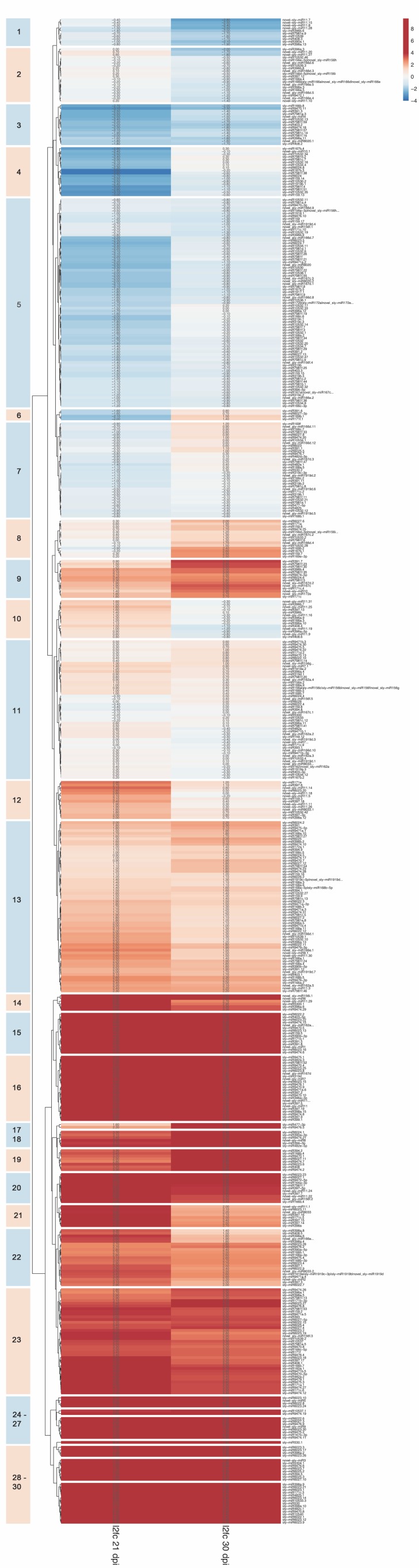
Expression profiles of miRNAs in tomato leaf tissues upon PVY^C^-to infection. The data matrix was subject to hierarchical clustering, and miRNAs were separated into 30 temporal expression patterns according to their (log_2_) fold change in PVY-infected vs. mock-inoculated tomato plants at 21 and 30 dpi. The expression level is shown in different colors, according to the heatmap on the right side: Red indicates upregulation, blue downregulation. Each row represents a miRNA.

In order to identify the miRNAs potentially involved in the regression of viral symptoms and replication at 30 dpi, differences in miRNA expression patterns at the two time points were examined. Hierarchical clustering was applied to the dataset to highlight the changes of miRNA expression profiles according to time course after PVY^C^-to infection, and miRNAs displaying similar expression patterns were grouped together (Figure 2, Table S4).

In clusters 3, 5, 9, 11, and 13–30, miRNAs showing similar levels of expression at both time points were grouped. Clear expression differences between 21 and 30 dpi were evident in two series of clusters, with the first series comprising clusters 1, 2, 10, and 12 including miRNAs whose accumulation levels decreased from 21 to 30 dpi. Notably, among the well-characterized miRNAs, sly-miR156 and sly-miR166 were negatively regulated only at 30 dpi, and sly-miR396a, sly-miR396b, and sly-miR397-3p exhibited higher accumulation at 21 dpi and then substantially decreased at 30dpi (Figure 2, Table S4). The second series (clusters 4, 6, 7, and 8) included miRNA with increasing accumulation from 21 to 30 dpi. Defense-related miRNA such as sly-miR482d-3p, sly-miR6023, sly-miR6024, and sly-miR6027-3p were predominantly downregulated at 21 dpi and increased their expression at the following time point, and members of the sly-miR156, sly-miR169, and sly-miR171 families were substantially upregulated at 30 dpi while their levels were not altered at 21 dpi (Figure 2, Table S4). 

Altogether, these results indicated that the effects of PVY^C^-to infection on miRNA metabolism declined from 21 to 30 dpi, and suggested that a set of miRNAs with changing expression pattern between time points could have an important biological effect in the transition from diseased to recovered conditions.

### 3.5. RT-qPCR Expression Analysis of Known and Novel miRNAs and Their Target mRNAs

**Figure 3:**
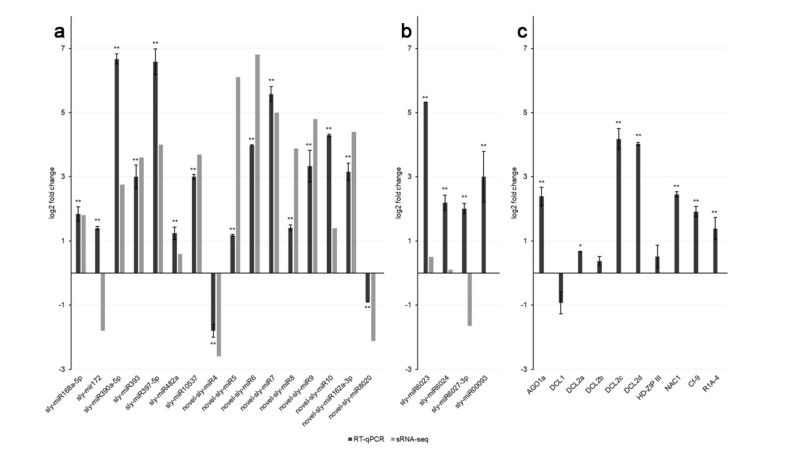
Expression levels of selected miRNAs and their mRNA targets in leaves of PVY^C^-to-infected tomato plants at 21 days post-inoculation (dpi). (**a**) Quantitative expression analysis of 16 tomato miRNAs upon PVY^C^-to infection. Black columns indicate miRNA expression levels determined by RT-qPCR, grey columns indicate miRNA expression levels determined by sRNA-seq; (**b**) RT-qPCR profiles of 4 miRNAs targeting *Cf-9* and *R1A-4* mRNAs in tomato; (**c**) Quantitative expression analysis of 10 miRNA target mRNAs upon PVY^C^-to infection. Columns indicate the mean gene expression levels of three biological replicates, determined by RT-qPCR [(log_2_) fold change in PVY-infected vs. healthy plants, error bars indicate standard errors]. Statistically significant differences between PVY-infected and healthy samples are indicated by asterisks (* P < 0.05, ** P < 0.01; Student’s t-test).

To confirm the sRNA-seq results, RT-qPCR expression analysis was performed on a randomly-selected subset of known and novel miRNAs, as well as on their target mRNAs. Thirteen miRNAs were upregulated and two were downregulated at 21 dpi according to both sRNA-seq and RT-qPCR, although fold change figures were different in some cases (Figure 3a). For one out of 16 miRNAs shown in Figure 3a, sly-miR172, RT-qPCR and sRNA-seq results for miRNA expression were discordant.

RT-qPCR also provided additional experimental evidence that 7 novel miRNAs were authentically expressed in tomato tissues. Novel-sly-miR4 was the only novel miRNA detected by sRNA-seq downregulated upon PVY infection, whereas 6 additional novel miRNAs were all significantly upregulated, as observed by sRNA-seq and confirmed by RT-qPCR (Figure 3a).

### 3.6. Target Genes Prediction and Functional Characterization of Differentially Expressed Tomato miRNAs

To better understand the role of miRNA-mediated pathways on host defense responses to PVY infection, we performed a functional analysis based on the known or the putative miRNA-regulated genes function. A total of 5867 unique miRNA/mRNA pairs were predicted by the psRNATarget tool, 1181 of which involved canonical miRNAs and the remaining part isomiRs (Table S5). We further tested whether the accumulation of selected miRNA-regulated transcripts in tomato could be altered as a consequence of PVY^C^-to ability to interfere with miRNA expression profiles. The comparison of the expression levels of the selected genes from infected and mock-inoculated plants at 21 dpi was made by RT-qPCR using the same leaf samples used for miRNA analysis (Figure 3). The abundance of *AGO1a*, *DCL2a*, *DCL2d*, and *NAC1* transcripts was significantly increased upon PVY infection compared with mock-inoculated plants (Figure 3c). *AGO1* is a target of sly-miR168a/b, *DCL2* family members are targets of sly-miR6026, and *NAC1* is a target of sly-miR164a/b. Thus, since the latter miRNAs were all upregulated in virus-infected plants (Table S1), in these four cases, a positive correlation between transcript and cognate miRNA accumulation levels was observed. On the other hand, the expression profiles of *DCL1* and *Homeobox leucine-zipper protein (HD-Zip III)*, and of their cognate miRNAs, sly-miR162 and sly-miR166a/b respectively, did not differ significantly upon viral infection, indicating that their gene expression homeostasis was not altered by PVY^C^-to (Figure 3c, Table S1). An unvaried level of sly-miR6026, as inferred by sRNA-seq data, corresponded to an increased accumulation of its targets *DCL2a* and *DCL2d*, whereas *DCL2b* did not show significant modulation (Figure 3c). 

We identified a list of predicted mRNA targets that were also for novel miRNAs (Table S7). We obtained a list of 148 putative targets since, as in the case of known miRNAs, for each individual novel miRNA, more than one (up to 27 for novel-sly-miR7) putative target were bioinformatically retrieved. Interestingly, three mRNAs coding for a Glycine-rich protein, a Disulfide-isomerase-like protein and a Protein phosphatase 2C family protein were identified as being post-transcriptionally regulated by novel-sly-miR1, novel-sly-miR6, and novel-sly-miR10 respectively, an outcome that was additionally supported by the results of the Degradome-seq analysis performed in silico on publicly-available datasets (Tables S7 and S9). Eight out of 15 putative targets of novel_sly-miR10 were NLRs, mitogen-activated protein kinases, or other resistance-related genes. Disease resistance genes were also predicted targets of other novel miRNAs (novel-sly-miR1, -miR4, -miR5, miR7, and –miR8). Finally, novel-sly-miR4, -miR5, -miR7, and –miR9 putatively targeted mRNAs classified in different transcription factor families (e.g. WRKY, MYB, ERF) (Table S7). 

**Figure 4:**
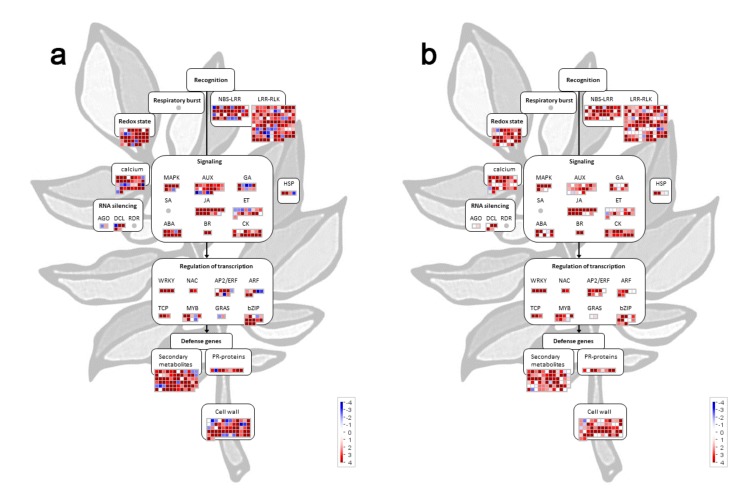
MapMan analysis showing differentially expressed miRNAs (DEMs) targeting multiple immune and hormone signaling components. Visualization of DEMs at (**a**) 20 dpi and (**b**) 30 dpi according to the function of their predicted targets. Each square represents (log_2_) fold change in PVY-infected vs. mock-inoculated tomato plants (red: upregulated; blue: downregulated).

To investigate the main biological pathways affected by the miRNA-mediated regulation, a MapMan analysis was performed based upon DEMs detected at 21 and 30 dpi and their predicted targets. The results revealed that miRNA which targets WRKYs and genes involved in jasmonic acid, ethylene, and brassinosteroid biosynthesis and signaling were concordantly upregulated at both time points, whereas miRNAs targeting nucleotide metabolism components were mostly downregulated (Table S6). When we focused on processes involved in plant response to biotic stress [61] (Figure 4), we discovered that several DEMs were targeting mRNAs coding for different immune receptors (NLRs, RLKs), RS proteins such as AGOs and DCLs, proteins maintaining redox homeostasis, proteins involved in biosynthesis and signaling of different phytohormones, calcium signaling, as well as transcription factors belonging to AP2/ERF, MYB, ARF, and bZIP family proteins were upregulated at 21 dpi and 30 dpi (Figure 4, Table S6). By closely inspecting the expression level of miRNAs targeting *NLRs* and *RLKs*, we observed that many miRNAs were commonly upregulated at both time points; however, several *NLR* and *RLKs*-targeting miRNAs exhibiting downregulation at 21 dpi were weakly downregulated or their level was unaltered at 30 dpi (Figure 4). The same trend was also observed for certain miRNAs targeting ARF and MYB transcription factors, auxin, ethylene, and gibberellin signaling components, as well as mRNAs encoding proteins involved in secondary metabolism, cell wall synthesis and degradation, and calcium signaling, suggesting that their diminished regulation could be important in the recovery process.

Therefore, our data indicate that PVY-infected tomato plants could activate and/or regulate via miRNA pathways several different defense responses mediated by NLRs and RLP/RLK receptors, transcription factors, RNA silencing, and other defense-related metabolic functions. The overall impact of PVY infection on the expression profiles of defense-related miRNAs was higher at 21 dpi and decreased at 30 dpi.

### 3.7. PVY^C^-to Infection Induces Secondary Phased siRNA Accumulation

miRNAs can potentiate their silencing capability by triggering the production of phasiRNAs from their targets [62]. Over 900 *PHAS* loci generating 21- or 24-nt phasiRNAs were identified (Table S8). The highest number (535) of *PHAS* loci was detected in PVY-21 library, and 180 out of these 535 loci were not detected in any of other libraries, whereas 63 *PHAS* loci were identified only in PVY libraries (Table S8). When inspecting the *PHAS* loci with the highest degree of phasing, we observed that the majority of them encoded immune receptor proteins harboring NB-ARC and/or LRR domains (Table S8). Next, we integrated 22-nt miRNAs, in silico predictions, and the Degradome-Seq data to identify the triggers for the predicted *PHAS* loci. MiRNA triggers for 114 and 38 *PHAS* loci were identified in silico and Degradome-Seq data, respectively (Table S8, Table S9), with a large number of predicted triggers belonging to the miR482, miR6023, miR6024, miR6025, and miR6027 families. For 19 interactions predicted in silico, the cleavage events were also detected by Degradome-Seq analysis (Table S8, Table S9). Different classes of immune receptors, including a majority of CC-NBS-LRRs (CNLs), but also TIR-NBS-LRRs (TNLs) and RLPs, were targeted for phasiRNA biogenesis in PVY-infected tomato plants (Figure S4, Table S12).

It is noteworthy that a restricted set of transcripts was predicted to be specifically targeted by isomiRs only. By limiting the search to disease-responsive genes (e.g. *NLRs*, *RLKs*, RS-related genes), 9 isomiRs, including 2 isomiRs from novel miRNAs, were specifically associated with a unique degraded transcript (Table S9). Three isomiRs’ predicted targets, i.e., *Solyc12g100030* (*RLP*), *Solyc01g008800* (*TNL*), and *Solyc11g008540* (*DCL2b*), were defense-related genes also found as phasiRNA-generating loci (Table S8), which strongly suggests that those genes could be miRNA/isomiR targets in PVY-infected tomato plants. Additionally, an isomiR, sly-miR6023.7, was the only 22-nt long isomiR in that set. Sly-miR6023.7 mapped with a +4 nt shift on the pre-miRNA sequence compared to mature sly-miR6023 and was found exclusively in PVY-infected sRNA libraries (Figure 1, Table 2). The degradome-seq analysis indicated four potential targets for sly-miR6023.7, including the *RLK Solyc09g064670* (Table S9).

To assess whether the virus infection can induce or suppress the phasiRNA production over the time of infection, the accumulation level of sRNAs mapping to *PHAS* loci was compared between PVY and mock sRNA samples at the two time points. By normalizing the four libraries to RPM and selecting genomic loci showing in-phase accumulation of secondary sRNAs (phase score >1), 241 and 140 loci gave origin to higher accumulation of sRNAs in PVY-infected samples than in mock samples at 21 and 30 dpi, respectively, displaying at least 2-fold higher sRNA accumulation. Of those *PHAS* loci, 54 were found to be upregulated at both time points, but 10 loci displayed an opposite regulation profile (i.e. phasiRNAs over-accumulated at 21 dpi and decreased accumulation at 30 dpi in PVY vs. healthy samples) (Figure S5). Eighty-nine and 84 loci showed higher phasiRNA accumulation in mock versus infected samples at 21 and 30 dpi respectively, with 22 loci being downregulated by PVY infection at both time points (Figure S5, Table S8).

Overall, these data indicate that cleavage by 22-nt miRNAs may trigger the production of phased secondary 21-nt siRNAs as a mechanism of fine regulation of defense-related gene expression that is dynamic over time, and that isomiRs are likely involved in the same mechanism in tomato plants infected by PVY^C^-to. 

### 3.8. miRNAs Mediate Cleavage of Transcripts Encoding NLRs and RLPs 

To analyze in more detail the activity of miRNAs, the phasiRNAs biogenesis and the post-transcriptional regulation of *RLP* and *NLR* gene expression during the PVY infection, we identified two *PHAS l*oci, i.e., *Solyc01g005870* and *Solyc05g008070*. 

**Figure 5:**
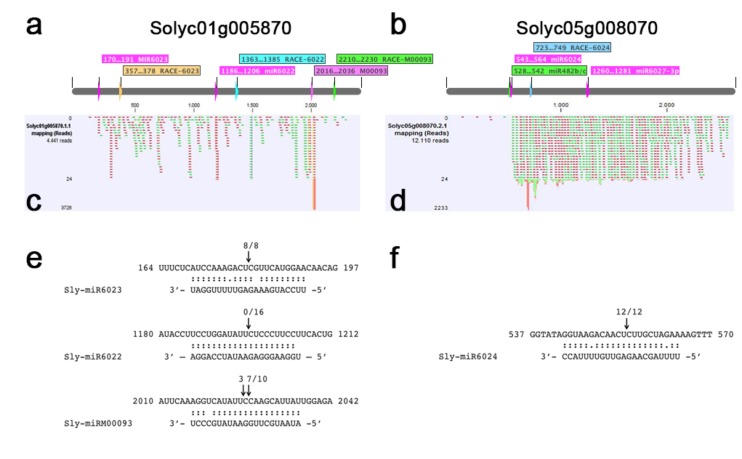
Transcripts of the tomato genes encoding Cf-9 (Solyc01g005870) and R1A-4 (Solyc05g008070) are targeted by miRNAs and generate phased secondary sRNAs in PVY-infected plants. (**a**,**b**) Diagram of *Cf-9/Solyc01g005870* (**a**) and *R1A-4/Solyc05g008070* (**b**) transcripts (not in scale), showing the predicted sites of miRNA cleavage (in pink) and the position of the gene-specific reverse primer for RLM 5’-RACE. (**c**,**d**) Mapping of secondary 21-nt sRNAs from the PVY-21 library (one mismatch allowed) on the *Cf-9/Solyc01g005870* (**c**) and *R1A-4/Solyc05g008070* (**d**) transcripts. Sense and antisense sRNAs are represented in green and red segments, respectively. On the left side, number of raw reads mapped at each position on the transcript. (**e**,**f**) Experimental validation of miRNA-mediated cleavage of predicted *R* gene targets by RLM 5’-RACE. miRNA-mRNA target perfect pairing (colon) and G:U wobble pairing (single point) are indicated. The arrows above the target mRNA indicate the number of independent clones that identified cleavage at that position

According to the NCBI *Solanum lycopersicum* Annotation Release 103, *Solyc01g005870* codes for the receptor-like protein Cf-9 [63] (NCBI Ref. Seq.: XM_004228576.4), and *Solyc05g008070* codes for a CNL, the putative late blight resistance protein homolog R1A-4 [64] (NCBI Ref. Seq.: XM_010322406). *Cf-9* was chosen for further analysis since, among sRNA reads mapping to its sequence, we found a very abundant (>3000 raw reads) 21-nt antisense sRNA in the PVY-21 library (Figure 5c). Searches in the Tomato Functional Genomics Database for small RNAs [65] identified a homologous miRNA named M00093 (Figure S6a). Although this miRNA family was neither retrieved in the miRBase nor identified as a novel miRNA by our own analysis, it was found in the literature, described as sly-miR00093 [66] or as tomato novel miRNA pc-27 [67]. Sly-miR00093 was predicted to target at least other seven *RLPs* which were closely related to *Cf-9* (Figure S6b). The same tomato transcript *Cf-9* was predicted also as the target of 22-nt-long sly-miR6023 and 21-nt-long sly-miR6022 by in silico and degradome-seq prediction analyses, respectively (Figure 5a, Table S8, Table S9). The second transcript, i.e., *R1A-4*, was the *NLR* gene with the third highest secondary sRNA accumulation level, and was predicted as the target of 22-nt sly-miR482b/c, sly-miR6024, sly-miR6026, and sly-miR6027-3p (Figure 5b, Table S8). At 21 dpi, a redundant set of 275 phasiRNA (81 RPM, phasescore = 4.4) mapped to *Cf-9*, while 1548 phasiRNA (456 RPM, phasescore = 25.2) mapped to *R1A-4* (Table S8).

miRNAs that were predicted to regulate *NLR* genes post-transcriptionally were quantified by RT-qPCR to further confirm the effects of viral infection on their accumulation levels. A general trend of upregulation was found upon PVY^C^-to infection at 21 dpi, as demonstrated for sly-miR6023, that was significantly overexpressed by 5.32 log_2_fc in infected vs. healthy tomato plants, for sly-miR6024 (log_2_fc = 2.18), for sly-miR6027-3p (log_2_fc = 2), and for sly-miR00093 (log_2_fc = 2.9) (Figure 3b). Divergence between sRNA-seq and RT-qPCR quantification could depend upon the fact that template RNA was extracted from two separate experiments, and could also be explained by the fact that these specific miRNAs, forward qPCR primers, can potentially detect and quantify more than one isomiR variant along with the canonical miRNA sequence (Table S10). 

To further verify the possibility that tomato miR00093, miR6022, and miR6023 concurred in the post-transcriptional regulation of *Cf-9*, we investigated the presence of specific cleavage products in PVY-infected tomato leaf tissues using RLM 5’-RACE. The predicted miRNA-guided cleavage products, corresponding to the position between nucleotides 10th and 11th of the miRNA sequences, were correctly amplified and sequenced in 8/8 PCR fragments for 22-nt sly-miR6023 and 7/11 PCR fragments for 21-nt sly-miR00093, and in the latter case, an additional cleavage site between nucleotides 11th and 12th of sly-miR00093 was identified in 3/11 PCR products (Figure 5e), indicating that both one-hit (22-nt miRNA) and two-hit (two 21-nt miRNA triggers) mechanisms were employed for the generation of phasiRNAs from *Cf-9.* In addition, the degradome-Seq data and results by Bai and colleagues [54] showed that *Cf-9* can be also targeted by miR6022 (Figure 5a). Since our RLM 5’-RACE experiment failed to detect any cleaved mRNA products at this specific site in PVY-infected tomato tissues (Figure 5e), our results suggest that the regulation of *Cf-9* by individual miRNAs might be disease-specific.

RLM 5’-RACE also validated the cleavage of *R1A-4*, position 554, as correctly predicted for the sly-miR6024-guided activity. In 12/12 cases, PCR fragments from RLM 5’-RACE evidenced a cleaved 5’-end which was compatible with this prediction (Figure 5f). Despite the demonstrated miRNA-guided cleavage activity on *Cf-9* and *R1A-4*, RT-qPCR analysis showed that both genes were overexpressed upon PVY^C^-to infection (Figure 3c). Therefore, the post-transcriptional expression regulation of these genes by miRNAs did not result in the straightforward suppression of gene accumulation in PVY-infected vs. healthy plants, but rather, in a sophisticated miRNA-triggered fine-tuning of gene expression, also involving the induction of phasiRNAs production from their targets. 

## 4. Discussion

In this study, we used a high-throughput sRNA-Seq approach to investigate the effects of PVY infection on sRNA metabolism and on regulation of immune responses in tomato. PVY infection in tomato induced a huge reprogramming of miRNA accumulation levels. The general effect of PVY^C^-to in infected tomato tissues was the upregulation of miRNAs. Similarly, a large predominance of upregulated miRNAs was also observed upon PVY infection in other hosts [13,28]. In this work, more than 200 miRNA sequences were shown to be consistently overexpressed at 21 and 30 dpi. Those can be considered as the “core” set of PVY-responsive miRNAs. Induction of miRNA expression is frequently regulated by transcription factors acting in trans on *MIR* gene-promoter sequences [68]. In virus-infected plants, an additional mechanism of miRNA accumulation depends on the presence of VSR proteins and their ability to bind sRNAs. HC-Pro, a VSR of PVY and other potyviruses, has been proven to bind miRNA/miRNA*, preventing miRNA loading into RISC and determining the abnormal accumulation of inactive miRNA and miRNA* forms [69,70,71]. The large number of over-accumulating miRNA* sequences found in our dataset indicates that the HC-Pro activity may be a major cause of alteration of miRNA metabolism also in PVY^C^-to- infected tomato plants. In fact, this observation implies that a conspicuous part of detected miRNA sequences is inactive as miRNA/miRNA* duplexes, and therefore, impaired in their gene expression regulatory function [69,70,72]. Accordingly, our work shows the occurrence in PVY-infected tomato plants of cases of: i) anomalous accumulation of miRNA* sequences, not detected in healthy plants (e.g. novel-sly-miR11*, miR164a-3p, miR168a-3p, miR171b-3p, miR396a-3p, etc.) and ii) positive correlation between accumulation levels of miRNAs and their target mRNAs.

Besides the quantitative effects on miRNA accumulation levels, PVY infection correlated with a remarkable increase of sequence variation within individual miRNA families. Although frequently underestimated, some authors warned about the importance of miRNA diversification and isomiR production in plant biology, since new isomiR biogenesis is proposed to strengthen or to fine-tune RS-based antiviral defenses [73,74]. Several enzymatic mechanisms have been shown to contribute to isomiR biogenesis (reviewed in [74]), some of which could be activated by virus infections possibly interfering with host immune responses. In rice for instance, severe infection caused by *Rice stripe virus*, but not mild infection caused by *Rice dwarf virus*, induced the accumulation of novel isomiR sequences from conserved pre-miRNAs. Those novel isomiRs were shown to regulate the expression of their predicted target mRNAs [75,76]. Novel miRNA variants were also observed by Hu and colleagues [77] in Arabidopsis plants infected with *Oilseed rape mosaic virus*. Our data indicate that many isomiRs are produced as a consequence of PVY^C^-to infection, and suggest that PVY-associated isomiRs such as sly-miR396a.2, sly-miR397.5, sly-miR6022.15, and sly-miR6023.7 have the potential to regulate the expression of *RLPs*, *NLRs* and RS-encoding genes by inducing mRNA cleavage and phasiRNA accumulation. To the best of our knowledge, our results represent the first example of the production of several different isomiRs strictly associated with the infection of a virus in a solanaceous host. Future research on this intriguing subject will expand our knowledge of the multifaceted role of miRNAs in plant defenses. 

MapMan analysis indicated that the altered expression of many miRNAs potentially affects the signaling and defense pathways involved in the plant response to biotic stresses. We showed that at 21 dpi during the acute phase of PVY infection, many defense gene-targeting miRNAs are downregulated, presumably allowing the increased expression of their respective targets that are deployed by host cells to counteract the pathogen. At 30 dpi, when decreased virus replication and alleviated symptoms were observed, the expression of defense genes could be relieved through the reduced alteration of miRNA levels (Figure 4). It is well known that many miRNA targets are transcription factors and genes regulating phytohormones biosynthesis, implying that plant miRNAs are master regulators of many physiological functions [78]. Following viral infection in plants, the regulation of transcription and hormone synthesis/signaling are often found among altered functional categories for miRNA targets [13,79]. Since many growth and development processes are controlled by miRNA-regulated factors, it has been proposed that the alteration of miRNA metabolism may represent one of the major mechanisms through which pathogenic viruses induce symptoms on infected plants [22,30,33,80].

Data from the present work also show significant changes in expression profiles of miRNAs controlling various classes of *R* genes including *NLRs* (*TIR-NBS-LRR* and *CC-NBS-LRR*), *RLPs,* and *RLKs*. Upon PVY infection, miR482, miR6022, miR6023, miR6024, and miR6027 families, known for targeting *R* genes in tomato [81,82,83], were found to be either up- or down- regulated in our libraries. Sly-miR6023 and sly-miR6024 were here validated as post-transcriptional regulators of *Cf-9* and *R1A-4*, respectively. An additional miRNA species, referred to as sly-miR00093, was also experimentally validated as another miRNA targeting *Cf-9* for post-transcriptional silencing. We provide evidence that miRNA-guided cleavage of *R* genes is a specific event which strictly depends on the host–pathogen combination: in fact, whereas *R1A-4* was proved to be a target for miR6024 in both PVY-infected (this work) and *Tomato yellow leaf curl Sardinia virus* (TYLCSV)-infected tomato cells [84], we failed to confirm the *Cf-9* cleavage operated by sly-miR6022, in contrast to what observed in *Tomato yellow leaf curl virus*-infected plants [54]. The low levels of mature sly-miR6022 in our libraries might explain the lack of cleavage, although sequence variations at miRNA recognition sites among host genotypes should also be taken into account as a possible reason for impaired miRNA activity [84]. 

The consequence of miRNA activity on target *R* genes is not only limited to mRNA cleavage, but can also lead to biogenesis of secondary, RDR6-dependent phasiRNAs [81,85]. *PHAS/R* loci discovery prompted scientists to suggest a mechanism by which *R* genes accumulation could be controlled by miRNAs by triggering secondary siRNA production finalized to potentiate post-transcriptional repression of resistance gene expression [86,87]. miRNA-mediated down-regulation of *NLRs* would allow the plant to balance in a dynamic process the metabolic costs of accumulating constitutively expressed NLR proteins and the benefits of effective protection against pests and pathogens [88,89]. The mechanism generates a “cascade” effect, since phasiRNAs would, in turn, amplify the defense response either by acting post-transcriptionally in cis, by silencing more *R* genes in trans [81], or by affecting the disease phenotype targeting other non-*R* genes [32,84]. Our data further corroborate the notion of phasiRNA-mediated defense mechanisms. In fact, the *NLR Solyc02g036270*, the *PHAS/R* locus with the highest degree of phasing in our study, and other loci such as *Solyc04g005540* and *R1A-4*, were also reported as secondary siRNA-producing loci in previous studies, [81,84,90]. However, the present work also provides new evidence that sheds light on this mechanism. Importantly, we show that a highly-significant phasiRNA biogenesis activity occurred at 21 dpi upon PVY infection, and therefore, correlated with the infection peak, whereas a more limited phasiRNA accumulation was observed at 30 dpi and in libraries from healthy plants. The additional observation that, at least for a limited number of *PHAS* loci, phasiRNA biogenesis can significantly vary over time from overexpression to suppression at an individual locus, suggests the existence of a highly-dynamic mechanism oriented towards the tight control of gene expression, which is particularly important for *NLR/RLP/RLK* genes. It is noteworthy that a reduced phasiRNA accumulation was observed at 30 dpi in comparison to 21 dpi, when infected leaf tissues exhibited a regression of symptoms. According to previous works, upon pathogen attack, plants respond with an enhanced *R* gene expression driven by suppressing the miRNA-mediated silencing pathway. The *PHAS/R* regulatory mechanism would be then activated for controlling excessive *R* gene expression [12,81]. More research is needed to refine a mechanistic model to explain phasiRNAs role in host recovery from PVY-induced diseases. 

Tomato genes encoding enzymes of the RS pathway were also found as phasiRNA-producing loci. It has been demonstrated that sly-miR6026 targets tomato DCL2a, DCL2b, and DCL2d in vivo, and that its cleavage is responsible for phasiRNA production at these loci [91]. In PVY-infected tomato, we could detect phasiRNA mapping on DCL2a and DCL2b, but not on DCL2d. Conversely, DCL2a and DCL2b did not show upregulation upon PVY infection, whereas DCL2c and DCL2d significantly increased. This observation suggests that DCL2 family members have redundant roles, and although Wang and colleagues (2018) indicated DCL2a/b as the major antiviral DCL2 forms, their post-transcriptional silencing could be replaced in infected tomato cells by increased levels of DCL2c/d. In fact, in *Tobacco mosaic virus*-infected *dcl2ab* tomato double mutants, the 22-nt viral sRNAs were less abundant but not absent, indicating residual DCL2 activity [91]. Apparently, HC-Pro, a VSR of PVY, suppresses RS in infected tomato plants without inhibiting the following process of secondary siRNA biogenesis where RDR6/SGS3 and DCL4 guide dsRNA synthesis and degradation, respectively. In contrast, TYLCSV RS suppressor, the V2 protein, was found to bind SGS3 and block antiviral silencing at the stage of single- to dsRNA polymerization operated by the RDR6/SGS3 complex. Probably for this reason, the production of phasiRNAs was abolished upon TYLCSV infection, and was only reported to occur in healthy tomato plants [84].

## 5. Conclusions

The work here presented confirms the existence of a wide range of endogenous sRNA-based responses encompassing quantitative miRNA alteration, qualitative diversification within miRNA families, and miRNA-driven secondary sRNA accumulation. From our investigation on host recovery from PVY-induced disease, we were able to identify a set of tomato sRNAs showing changes in accumulation levels over time, between 21 and 30 dpi. Although the molecular mechanisms leading to disease symptom suppression is still unknown, the intriguing possibility that recovery from viral infections may be supported by the direct involvement of miRNAs and other endogenous sRNA has been already suggested by other authors [92] and needs to be further explored. Suggestions of a putative role of these mechanisms in plant defenses and immunity are valuable, since they will provide the basis to reveal further plant–virus interactions, and will contribute to the development of control measures against viral diseases.

## Figures and Tables

**Table 1:** Overview of high-throughput sequencing results: classification of sRNAs from virus-infected and mock-inoculated tomato sRNA libraries at 21 and 30 dpi.

	**Mk-21 ^1^**		**PVY-21 ^1^**		**Mk-30 ^1^**		**PVY-30 ^1^**	
	**Redundant Reads Number**	**Nonredundant Reads Number**	**Redundant Reads Number**	**Nonredundant Reads Number**	**Redundant Reads Number**	**Nonredundant Reads Number**	**Redundant Reads Number**	**Nonredundant Reads Number**
raw reads (library size)	28.222.339	6.959.896	23.645.346	3.744.089	17.602.784	4.531.582	12.801.610	3.164.947
low-complexity sequences	195	171	105	93	172	148	134	118
clean reads 18–26 nt	22.463.045	6.376.853	21.507.027	3.392.422	13.973.890	4.137.134	10.945.324	2.845.983
**Length Distribution of Clean Reads**	**Redundant Reads Number**	**% ^2^**	**Redundant Reads Number**	**% ^2^**	**Redundant Reads Number**	**% ^2^**	**Redundant Reads Number**	**% ^2^**
18	207.038	0.92	201.139	0.94	151.081	1.08	137.259	1.25
19	280.484	1.25	288.476	1.34	175.548	1.26	182.771	1.67
20	495.843	2.21	484.206	2.25	300.467	2.15	281.912	2.58
**21**	**2.133.848**	**9.5**	**12.018.765**	**55.88**	**1.661.996**	**11.89**	**4.418.744**	**40.37**
**22**	**2.226.996**	**9.91**	**3.473.057**	**16.15**	**1.622.666**	**11.61**	**1.679.439**	**15.34**
**23**	**3.475.721**	**15.47**	**1.266.933**	**5.89**	**2.060.721**	**14.75**	**1.018.064**	**9.3**
**24**	**12.562.212**	**55.92**	**3.488.376**	**16.22**	**7.372.172**	**52.76**	**2.930.233**	**26.77**
25	761.854	3.39	197.821	0.92	425.439	3.04	198.083	1.81
26	319.049	1.42	88.254	0.41	203.800	1.46	98.819	0.9
**tomato SL3.0 genome mapped reads (100% identity)**	20.289.212	90.32	10.134.178	47.12	12.632.820	90.40	6.506.699	59.45
**noncoding RNAs**	**1787544**	**7.96**	**558036**	**2.59**	**1157563**	**8.28**	**554155**	**5.06**
rRNAs	1653355	7.36	519130	2.41	1034369	7.40	516761	4.72
tRNAs	73891	0.33	25743	0.12	95440	0.68	22052	0.20
snoRNA	48703	0.22	6736	0.03	20753	0.15	10516	0.10
snRNA	6507	0.03	3602	0.02	4221	0.03	2674	0.02
lncRNA	5088	0.02	2825	0.01	2780	0.02	2152	0.02
Tot.	**1787544**	**7.96**	**558036**	**2.59**	**1157563**	**8.28**	**554155**	**5.06**
**tomato phasiRNAs all**	53902	0.27	50163	0.49	20991	0.17	19981	0.31
**tomato phasiRNAs PVY specific**	0	0	313	0.003	0	0	219	0.003
**known mature miRNAs**	758288	3.38	883482	4.11	810148	5.80	455002	4.16
**vsiRNAs (100% identity)**	928	0.004	10040027	46.68	590	0.004	3593569	32.83
sequence length of vsiRNAs	**abs**	**% ^3^**	**abs**	**% ^3^**	**abs**	**% ^3^**	**abs**	**% ^3^**
18	2	0.22	17649	0.18			7299	0.20
19	5	0.54	24487	0.24	1	0.17	9812	0.27
20	9	0.97	66285	0.66	5	0.85	24028	0.67
**21**	**726**	**78.23**	**8209564**	**81.77**	**469**	**79.49**	**2914094**	**81.09**
**22**	**179**	**19.29**	**1652385**	**16.46**	**109**	**18.47**	**616990**	**17.17**
23	4	0.43	41968	0.42	4	0.68	13838	0.39
24	3	0.32	24511	0.24	2	0.34	6138	0.17
25			2405	0.02			984	0.03
26			773	0.01			386	0.01

^1^: small RNA libraries from: (1) Mk-21, mock-inoculated tomato plants at 21 days post-inoculation (dpi); (2) PVY-21, PVYC-to-inoculated tomato plants at 21 dpi; (3) Mk-30, mock-inoculated tomato plants at 30 dpi; 2) PVY-30, PVYC-to-inoculated tomato plants at 30 dpi. ^2^: percentage of redundant clean reads 18–26 nt in the corresponding library ^3^: percentage of vsiRNA (100% identity) in the corresponding library.

**Table 2:** Partial dataset from the sRNA-Seq analysis, showing normalized read counts and expression profiles of 31 isomiRs mapping to the tomato miR6023 precursor. Quantitatively predominant miRNAs in each cluster are indicated in bold.

			Normalized Read Counts (RPM)				Relative Accumulation (PVY vs. Mk)		
Cluster ^1^	Length	miRNA ID	Mk-21	PVY-21	Mk-30	PVY-30	Log_2_fc 21 dpi	Log_2_fc 30 dpi	PVY-Associated ^2^
**1**	**22**	**sly-miR6023**	**257.9**	**161.2**	**118.1**	**177.5**	**−0.7**	**0.6**	
1	23	sly-miR6023.30	4.3	154.3	1.3	185.2	5.2	7.2	
1	19	sly-miR6023.3	0.2	46.1	0.1	31.0	7.9	9.0	
1	23	sly-miR6023.14	0.0	4.1	0.1	4.5	6.8	5.2	YES
1	23	sly-miR6023.16	0.1	2.9	0.1	5.6	4.3	6.5	YES
1	22	sly-miR6023.7	0.0	2.6	0.0	2.5	7.1	6.4	YES
1	22	sly-miR6023.13	0.1	1.7	0.0	1.1	4.5	5.2	YES
1	22	sly-miR6023.21	0.0	1.7	0.0	2.5	6.5	6.4	YES
1	21	sly-miR6023.29	0.7	0.5	0.4	0.6			
**2**	**22**	**sly-miR6023.1**	**0.6**	**59.3**	**0.6**	**51.0**	**6.6**	**6.4**	
2	21	sly-miR6023.4	0.2	1.4	0.2	1.1	2.9	2.7	
2	24	sly-miR6023.5	3.4	0.6	2.3	1.3	−2.4	−0.8	
**3**	**22**	**sly-miR6023.28**	**13.5**	**87.2**	**13.9**	**96.9**	**2.7**	**2.8**	
3	21	sly-miR6023.18	0.7	15.3	1.5	12.0	4.4	3.0	
3	21	sly-miR6023.17	0.1	14.8	0.0	9.1	7.6	8.2	YES
3	22	sly-miR6023.10	0.1	9.0	0.0	12.2	6.3	8.7	YES
3	21	sly-miR6023.19	0.4	6.3	0.8	5.2	3.8	2.7	
3	21	sly-miR6023.27	0.6	5.8	0.5	8.9	3.4	4.1	
3	21	sly-miR6023.25	0.1	5.3	0.1	6.1	5.6	5.7	YES
3	21	sly-miR6023.2	0.3	3.8	0.5	3.6	3.7	2.9	
3	22	sly-miR6023.26	0.0	3.6	0.0	6.0	7.6	7.7	YES
3	23	sly-miR6023.11	0.0	1.3	0.3	1.3	5.1	2.1	YES
**4**	**21**	**sly-miR6023.15**	**0.1**	**2.2**	**0.1**	**4.2**	**4.9**	**5.1**	**YES**
4	21	sly-miR6023.12	0.0	1.4	0.0	1.1	6.2	5.1	YES
**5**	**19**	**sly-miR6023.23**	**1.6**	**43.1**	**2.6**	**47.7**	**4.7**	**4.2**	
5	20	sly-miR6023.24	0.1	5.9	0.0	7.1	5.3	7.9	YES
5	20	sly-miR6023.8	0.1	5.4	0.1	6.3	5.6	5.7	YES
5	18	sly-miR6023.22	0.1	2.4	0.1	2.0	4.0	5.1	YES
5	21	sly-miR6023.20	0.3	2.0	1.1	1.5	2.8	0.4	
5	22	sly-miR6023.6	0.1	1.3	0.0	1.5	3.6	5.7	YES
5	21	sly-miR6023.9	0.0	1.3	0.1	2.3	6.2	5.2	YES

^1^: Clusters 1–5 as described in Figure 1; ^2^: Not or barely detectable (RPM ≤ 0.1) in both Mk-21 and Mk-30 libraries.

**Table 3:** Differentially expressed tomato canonical tomato miRNAs upon PVY^C^-to infection at 21 and 30 dpi.

				Normalized Read Counts (RPM)				Relative Accumulation (PVY vs. Mk)	
Sequence	miRNA ID ^1^	Identical miRNA ID in miRBase ^2^	miRNA Family	Mk-21	PVY-21	Mk-30	PVY-30	Log_2_fc 21 dpi	Log_2_fc 30 dpi
GGAGGCAGCGGTTCATCGATC	novel_sly-miR162a*	aly-miR162a-5p	miR162	6.2	130.8	3.2	96.9	4.4	4.9
AGATCATGTGGTTGCTTCACC	novel_sly-miR167d	NO	miR167	1.0	36.0	0.5	30.1	5.1	5.8
TTCCAAAGCTGCAGAAATGAGT	novel_sly-miR8033	stu-miR8033-5p	miR8033	1.2	17.8	2.8	12.7	3.9	2.2
TTTTGTAGTAACTGTACCACA	novel-sly-miR1	NO	novel	0.1	2.9	0.0	2.3	4.3	6.2
GGGGCAACTTGAGATCACATG	novel-sly-miR11	NO	novel	295.5	19,873	184.5	4511.7	6.1	4.6
TGTGTTCTCAGGTTACCCCTG	novel-sly-miR11*	NO	novel	254.1	9302.3	187.6	4058.3	5.2	4.4
AACTGTCGGGAGACATTAGCT	novel-sly-miR2	NO	novel	1.1	8.4	1.6	7.1	2.9	2.2
TACTATCTGATTTAAGATTAG	novel-sly-miR3	NO	novel	0.0	4.5	0.0	3.6	7.9	6.9
CCTGAACTATCACCATCTATG	novel-sly-miR5	NO	novel	0.1	7.8	0.0	5.3	6.1	7.5
TGGCAAGTAAGCGCTCCAACT	novel-sly-miR6	NO	novel	0.0	4.3	0.2	3.8	6.8	4.4
AGGGGAGATAGATGAAGTTAGG	novel-sly-miR7	NO	novel	0.2	7.3	0.2	7.1	5.0	5.3
TTATTTGGGGTAGATGAGCTC	novel-sly-miR8	NO	novel	1.0	14.3	0.4	9.5	3.9	4.7
AACAACTGATAGTTGAGGTGT	novel-sly-miR9	NO	novel	0.3	7.5	0.0	4.8	4.8	7.3
ATTTACCCCAAGTTCGTTGTC	sly-miR10537	sly-miR10537	miR10537	1.2	14.1	0.9	8.4	3.6	3.2
ATAATAACTATTAGTTGAATG	sly-miR10540	sly-miR10540	miR10540	0.0	1.5	0.0	1.2	6.3	5.3
CATGTGCCTGTTTTCCCCATC	sly-miR164a-3p	sly-miR164a-3p	miR164	0.9	30.8	1.0	14.9	5.1	4.0
GGGATGTTGTCTGGCTCGACA	sly-miR166c-5p	sly-miR166c-5p	miR166	155.7	1440.6	99.9	905.8	3.2	3.2
AGGTCATCTAGCAGCTTCAAT	sly-miR167b-3p	sly-miR167b-3p	miR167	0.2	9.5	0.1	7.0	5.4	6.9
CCTGCCTTGCATCAACTGAAT	sly-miR168a-3p	sly-miR168a-3p	miR168	67.3	441.4	32.9	201.2	2.7	2.6
CCCGCCTTGCATCAACTGAAT	sly-miR168b-3p	sly-miR168b-3p	miR168	220.1	1731.6	121.5	794.4	3.0	2.7
TTGAGCCGTGCCAATATCACG	sly-miR171b-3p	sly-miR171b-3p	miR171	3.4	28.0	1.4	22.0	3.0	4.0
TATTGGCCTGGTTCACTCAGA	sly-miR171f	sly-miR171f	miR171	36.6	371.0	25.1	226.4	3.3	3.2
ACGAGAGTCATCTGTGACAGG	sly-miR1919a|sly-miR1919c-3p|sly-miR1919b|novel_sly-miR1919d	sly-miR1919a	miR1919	9.0	93.2	9.0	52.8	3.4	2.6
AGGAAACTGTTTAGTCCAACC	sly-miR319d	sly-miR319d	miR319	0.0	1.2	0.0	1.6	5.0	5.8
CGCTATCCATCCTGAGTTTTA	sly-miR390a-3p	sly-miR390a-3p	miR390	0.5	3.9	0.2	6.1	3.0	4.7
AAGCTCAGGAGGGATAGCACC	sly-miR390a-5p	sly-miR390a-5p	miR390	75.9	524.5	70.9	378.0	2.8	2.4
CGCTATCCATCCTGAGTTTCA	sly-miR390b-3p	sly-miR390b-3p	miR390	0.1	1.6	0.0	1.1	4.4	5.2
ATCATGCGATCTCTTCGGAAT	sly-miR393	sly-miR393	miR393	10.0	120.2	6.7	76.1	3.6	3.5
AGGTGGGCATACTGTCAACA	sly-miR394-3p	sly-miR394-3p	miR394	1.3	16.4	1.6	28.5	3.7	4.2
GTTCAATAAAGCTGTGGGAAG	sly-miR396a-3p	sly-miR396a-3p	miR396	49.9	1727.2	45.5	1132.1	5.1	4.6
ATTGAGTGCAGCGTTGATGA	sly-miR397-5p	sly-miR397-5p	miR397	10.0	359.3	15.5	208.7	5.2	3.7
TATGTTCTCAGGTCGCCCCTG	sly-miR398a	sly-miR398a	miR398	834.8	13869	925.1	5806.1	4.1	2.6
CGTTTGTGCGTGAATCTAACA	sly-miR403-5p	sly-miR403-5p	miR403	0.7	11.9	0.2	9.7	4.1	5.3
ACGGGGACGAGCCAGAGCATG	sly-miR408	sly-miR408	miR408	1.6	14.5	1.0	40.2	3.2	5.4
TGTGGGTGGGGTGGAAAGATT	sly-miR482e-5p	sly-miR482e-5p	miR482	6.0	89.3	9.6	202.5	3.9	4.4
AGGTGTAGGTGTTCATGCAGA	sly-miR530	sly-miR530	miR530	0.4	48.7	0.4	18.7	6.8	5.7
ATGGGTAGCACAAGGATTAATG	sly-miR6027-5p	sly-miR6027-5p	miR6027	313.9	4006.6	281.9	3442.1	3.7	3.6
TGAAATCCATGAGCCTAAACT	sly-miR9470-5p	sly-miR9470-5p	miR9470	0.7	13.6	0.5	5.3	4.2	3.5
TTTCAGTAGACGTTGTGAATA	sly-miR9472-5p	sly-miR9472-5p	miR9472	0.2	4.3	0.2	2.9	4.5	4.0
TGTAGAAGTCATGAATAAAATG	sly-miR9474-5p	sly-miR9474-5p	miR9474	6.3	26.5	6.0	33.6	2.1	2.5
AAAAAGATGCAGGACTAGACC	sly-miR9476-3p	sly-miR9476-3p	miR9476	114.0	675.9	112.5	493.2	2.6	2.1
AACAACATACTTACTGAAATGCCA	novel_sly-miR8020	NO	miR8020	6.6	1.3	5.5	2.7	−2.3	−1.0
GAATTTCATTGAGTATGTTGTTGT	novel_sly-miR8020*	NO	miR8020	1.6	0.3	0.6	0.1	−2.6	−
AGTGGACAAGTAAAGGTGGATGGA	novel-sly-miR4	NO	novel	5.2	0.9	3.3	1.1	−2.6	−1.5
AACGAGTGAGACTTGCTCAGTTGG	sly-miR10529	sly-miR10529	miR10529	1.6	0.3	0.4	0.8	−2.6	−
ACGTCCCTTCCCCATCGTTCAACA	sly-miR10530	sly-miR10530	miR10530	3.7	0.7	2.7	1.3	−2.3	−1.1
TTTTAGCAAGAGTTGTTTTACC	sly-miR6024	sly-miR6024	miR6024	365.2	56.1	158.0	90.1	−2.7	−0.8
AAGTGTGTCTCTGGAATTTCGGGC	sly-miR7981f	sly-miR7981f	miR7981	5.2	1.1	3.4	2.5	−2.2	−0.4

^1^: prefix novel_ denotes miRNAs originating from newly discovered tomato *MIR* genes belonging to known miRNA families; prefix novel- denotes the newly identified tomato miRNAs from newly discovered tomato *MIR* genes classified into novel miRNA families. ^2^: for previously described miRNAs, the identifier (ID) of the identical miRNAs in miRBase is also given.

## Supplementary Materials

The following are available online at https://www.mdpi.com/1999-4915/11/12/1100/s1. Figure S1. PVYC-to-infected tomato plants exhibit disease recovery at 30 dpi. Figure S2. Classification of miRNA and isomiR sequences found in libraries from mock-inoculated and PVYC-to-infected tomato leaf tissues. Figure S3. Venn diagrams showing the overlap of DEMs between 21 and 30 dpi. Figure S4. Phylogenetic tree of LRR-domain-containing tomato NLR or RLP coding sequences. Figure S5. Venn diagrams showing the overlap of PHAS loci showing increase or suppression of phasiRNAs accumulation in PVY-infected vs. mock-inoculated plants at 21 and 30 dpi. Figure S6. Characterization of sly-miR00093. Table S1. miRNAs identified in PVY- and mock-inoculated tomato samples together with their expression levels. Table S2. Novel MIR loci and miRNAs identified in PVY- and mock-inoculated tomato plants. Table S3. List of miRNAs and isomiRs specifically associated with PVY-infected plants. Table S4. Clustering results based on the expression profiles of mature miRNAs and isomiRs upon PVYC-to infection at 21 and 30 dpi. Table S5. Predicted targets of miRNAs by in silico approach. Table S6. MapMan analysis results. Table S7. Predicted targets of novel miRNAs by in silico approach. Table S8. Identified PHAS loci. Table S9. Bioinformatic validation of mRNA targets of tomato miRNAs by Degradome-Seq. Table S10. Quantification of miR6023, miR6024 and miR6027 by sRNA-Seq and RT-qPCR. Table S11. List of primers used in RT-qPCR and 5′ RACE experiments in this work. Table S12. List of the 20 disease-related genes encoding NLRs and transmembrane RLP/RLK receptors, selected by the capability to generate phasiRNAs.

## References

[1] D’Ario M., Griffiths-Jones S., Kim M. (2017). Small RNAs: Big impact on plant development. Trends Plant Sci..

[2] Baulcombe D.C., Dean C. (2014). Epigenetic regulation in plant responses to the environment. Cold Spring Harb. Perspect. Biol..

[3] Axtell M.J. (2013). Classification and comparison of small RNAs from plants. Annu. Rev. Plant Biol..

[4] Li F., Pignatta D., Bendix C., Brunkard J.O., Cohn M.M., Tung J., Sun H., Kumar P., Baker B. (2012). MicroRNA regulation of plant innate immune receptors. Proc. Natl. Acad. Sci. USA.

[5] Yang L., Huang H. (2014). Roles of small RNAs in plant disease resistance. J. Integr. Plant Biol..

[6] Fei Q., Zhang Y., Xia R., Meyers B.C. (2016). Small RNAs Add Zing to the Zig-Zag-Zig Model of Plant Defenses. Mol. Plant-Microbe Interact..

[7] Huang J., Yang M., Zhang X. (2016). The function of small RNAs in plant biotic stress response. J. Integr. Plant Biol..

[8] Yang Z., Li Y. (2018). Dissection of RNAi-based antiviral immunity in plants. Curr. Opin. Virol..

[9] Meng Y., Shao C., Wang H., Chen M. (2011). The Regulatory Activities of Plant MicroRNAs: A More Dynamic Perspective. Plant Physiol..

[10] Bai M., Yang G.S., Chen W.T., Mao Z.C., Kang H.X., Chen G.H., Yang Y.H., Xie B.Y. (2012). Genome-wide identification of Dicer-like, Argonaute and RNA-dependent RNA polymerase gene families and their expression analyses in response to viral infection and abiotic stresses in Solanum lycopersicum. Gene.

[11] Wang X.B., Wu Q., Ito T., Cillo F., Li W.X., Chen X., Yu J.L., Ding S.W. (2010). RNAi-mediated viral immunity requires amplification of virus-derived siRNAs in Arabidopsis thaliana. Proc. Natl. Acad. Sci. USA.

[12] Deng P., Muhammad S., Cao M., Wu L. (2018). Biogenesis and regulatory hierarchy of phased small interfering RNAs in plants. Plant Biotechnol. J..

[13] Križnik M., Petek M., Dobnik D., Ramsak Z., Baebler S., Pollmann S., Kreuze J.F., Zel J., Gruden K. (2017). Salicylic Acid Perturbs sRNA-Gibberellin Regulatory Network in Immune Response of Potato to Potato virus Y Infection. Front. Plant Sci..

[14] Fei Q., Xia R., Meyers B.C. (2013). Phased, secondary, small interfering RNAs in posttranscriptional regulatory networks. Plant Cell.

[15] Ding S.-W., Voinnet O. (2007). Antiviral immunity directed by small RNAs. Cell.

[16] Baulcombe D. (2004). RNA silencing in plants. Nature.

[17] Stavolone L., Prigigallo M.I., Cillo F., Palmiro P., Yiguo H. (2019). Plant viruses against RNA silencing based defenses: Strategies and solutions. Applied Plant Biotechnology for Improving Resistance to Biotic Stress.

[18] Csorba T., Kontra L., Burgyán J. (2015). Viral silencing suppressors: Tools forged to fine-tune host-pathogen coexistence. Virology.

[19] Kasschau K.D., Xie Z., Allen E., Llave C., Chapman E.J., Krizan K.A., Carrington J.C. (2003). P1/HC-Pro, a viral suppressor of RNA silencing, interferes with Arabidopsis development and miRNA function. Dev. Cell.

[20] Lewsey M., Robertson F.C., Canto T., Palukaitis P., Carr J.P. (2007). Selective targeting of miRNA-regulated plant development by a viral counter-silencing protein. Plant J..

[21] Díaz-Pendón J.A., Ding S.-W. (2008). Direct and indirect roles of viral suppressors of RNA silencing in pathogenesis. Annu. Rev. Phytopathol..

[22] Cillo F., Mascia T., Pasciuto M.M., Gallitelli D. (2009). Differential effects of mild and severe Cucumber mosaic virus strains in the perturbation of MicroRNA-regulated gene expression in tomato map to the 3’ sequence of RNA 2. Mol. Plant Microbe Interact..

[23] Ghoshal B., Sanfacon H. (2015). Symptom recovery in virus-infected plants: Revisiting the role of RNA silencing mechanisms. Virology.

[24] Nie X., Molen T.A. (2015). Host recovery and reduced virus level in the upper leaves after Potato virus Y infection occur in tobacco and tomato but not in potato plants. Viruses.

[25] Scholthof K.B.G., Adkins S., Czosnek H., Palukaitis P., Jacquot E., Hohn T., Hohn B., Saunders K., Candresse T., Ahlquist P. (2011). Top 10 plant viruses in molecular plant pathology. Mol. Plant Pathol..

[26] Lorenzen J.H., Meacham T., Berger P.H., Shiel P.J., Crosslin J.M., Hamm P.B., Kopp H. (2006). Whole genome characterization of Potato virus Y isolates collected in the western USA and their comparison to isolates from Europe and Canada. Arch. Virol..

[27] Ogawa T., Tomitaka Y., Nakagawa A., Ohshima K. (2008). Genetic structure of a population of Potato virus Y inducing potato tuber necrotic ringspot disease in Japan; comparison with North American and European populations. Virus Res..

[28] Guo Y., Jia M.A., Yang Y., Zhan L., Cheng X., Cai J., Zhang J., Yang J., Liu T., Fu Q. (2017). Integrated analysis of tobacco miRNA and mRNA expression profiles under PVY infection provids insight into tobacco-PVY interactions. Sci. Rep..

[29] Diermann N., Matousek J., Junge M., Riesner D., Steger G. (2010). Characterization of plant miRNAs and small RNAs derived from potato spindle tuber viroid (PSTVd) in infected tomato. Biol. Chem..

[30] Feng J., Liu S., Wang M., Lang Q., Jin C. (2014). Identification of microRNAs and their targets in tomato infected with Cucumber mosaic virus based on deep sequencing. Planta.

[31] Pradhan B., Naqvi A.R., Saraf S., Mukherjee S.K., Dey N. (2015). Prediction and characterization of Tomato leaf curl New Delhi virus (ToLCNDV) responsive novel microRNAs in Solanum lycopersicum. Virus Res..

[32] Zheng Y., Wang Y., Ding B., Fei Z. (2017). Comprehensive Transcriptome Analyses Reveal that Potato Spindle Tuber Viroid Triggers Genome-Wide Changes in Alternative Splicing, Inducible trans-Acting Activity of Phased Secondary Small Interfering RNAs, and Immune Responses. J. Virol..

[33] Tripathi A., Goswami K., Tiwari M., Mukherjee S.K., Sanan-Mishra N. (2018). Identification and comparative analysis of microRNAs from tomato varieties showing contrasting response to ToLCV infections. Physiol. Mol. Biol. Plants.

[34] Mascia T., Finetti-Sialer M., Cillo F., Gallitelli D. (2010). Biological and molecular characterization of a recombinant isolate of potato virus Y associated with a tomato necrotic disease occurring in Italy. J. Plant Pathol..

[35] Mascia T., Santovito E., Gallitelli D., Cillo F. (2010). Evaluation of reference genes for quantitative reverse-transcription polymerase chain reaction normalization in infected tomato plants. Mol. Plant Pathol..

[36] Martin M. (2011). Cutadapt removes adapter sequences from high-throughput sequencing reads. EMBnet J..

[37] Moxon S., Schwach F., Dalmay T., Maclean D., Studholme D.J., Moulton V. (2008). A toolkit for analysing large-scale plant small RNA datasets. Bioinformatics.

[38] Bateman A., Agrawal S., Birney E., Bruford E.A., Bujnicki J.M., Cochrane G., Cole J.R., Dinger M.E., Enright A.J., Gardner P.P. (2011). RNAcentral: A vision for an international database of RNA sequences. RNA.

[39] Kozomara A., Griffiths-Jones S. (2014). miRBase: Annotating high confidence microRNAs using deep sequencing data. Nucleic Acids Res..

[40] Shahid S., Axtell M.J. (2014). Identification and annotation of small RNA genes using ShortStack. Methods.

[41] Lei J., Sun Y. (2014). miR-PREFeR: An accurate, fast and easy-to-use plant miRNA prediction tool using small RNA-Seq data. Bioinformatics.

[42] Langmead B., Salzberg S.L. (2012). Fast gapped-read alignment with Bowtie 2. Nat. Methods.

[43] Huang Y., Niu B., Gao Y., Fu L., Li W. (2010). CD-HIT Suite: A web server for clustering and comparing biological sequences. Bioinformatics.

[44] De Oliveira L.F.V., Christoff A.P., Margis R. (2013). isomiRID: A framework to identify microRNA isoforms. Bioinformatics.

[45] Neilsen C.T., Goodall G.J., Bracken C.P. (2012). IsomiRs—the overlooked repertoire in the dynamic microRNAome. Trends Genet..

[46] Metsalu T., Vilo J. (2015). ClustVis: A web tool for visualizing clustering of multivariate data using Principal Component Analysis and heatmap. Nucleic Acids Res..

[47] Dai X., Zhuang Z., Zhao P.X. (2018). psRNATarget: A plant small RNA target analysis server (2017 release). Nucleic Acids Res..

[48] Cuperus J.T., Carbonell A., Fahlgren N., Garcia-Ruiz H., Burke R.T., Takeda A., Sullivan C.M., Gilbert S.D., Montgomery T.A., Carrington J.C. (2010). Unique functionality of 22-nt miRNAs in triggering RDR6-dependent siRNA biogenesis from target transcripts in Arabidopsis. Nat. Struct. Mol. Biol..

[49] Chen H.-M., Chen L.-T., Patel K., Li Y.-H., Baulcombe D.C., Wu S.-H. (2010). 22-nucleotide RNAs trigger secondary siRNA biogenesis in plants. Proc. Natl. Acad. Sci. USA.

[50] Hall T.A. (1999). BioEdit: A user-friendly biological sequence alignment editor and analysis program for Windows 95/98/NT. Nucleic Acids Symp. Ser..

[51] Saitou N., Nei M. (1987). The neighbor-joining method: A new method for reconstructing phylogenetic trees. Mol. Biol. Evol..

[52] Kumar S., Stecher G., Li M., Knyaz C., Tamura K. (2018). MEGA X: Molecular Evolutionary Genetics Analysis across Computing Platforms. Mol. Biol. Evol..

[53] Usadel B., Poree F., Nagel A., Lohse M., Czedik-Eysenberg A., Stitt M. (2009). A guide to using MapMan to visualize and compare Omics data in plants: A case study in the crop species, Maize. Plant Cell Environ..

[54] Bai M., Yang G.S., Chen W.T., Lin R.M., Ling J., Mao Z.C., Xie B.Y. (2016). Characterization and function of Tomato yellow leaf curl virus-derived small RNAs generated in tolerant and susceptible tomato varieties. J. Integr. Agric..

[55] Addo-Quaye C., Miller W., Axtell M.J. (2009). CleaveLand: A pipeline for using degradome data to find cleaved small RNA targets. Bioinformatics.

[56] Addo-Quaye C., Eshoo T.W., Bartel D.P., Axtell M.J. (2008). Endogenous siRNA and miRNA targets identified by sequencing of the Arabidopsis degradome. Curr. Biol..

[57] Llave C., Xie Z., Kasschau K.D., Carrington J.C. (2002). Cleavage of Scarecrow-like mRNA targets directed by a class of Arabidopsis miRNA. Science.

[58] Xu P., Billmeier M., Mohorianu I.-I., Green D., Fraser W., Dalmay T. (2015). An improved protocol for small RNA library construction using high definition adapters. Methods Next Gener. Seq..

[59] Corpet F. (1988). Multiple sequence alignment with hierarchical clustering. Nucleic Acids Res..

[60] Cloonan N., Wani S., Xu Q., Gu J., Lea K., Heater S., Barbacioru C., Steptoe A.L., Martin H.C., Nourbakhsh E. (2011). MicroRNAs and their isomiRs function cooperatively to target common biological pathways. Genome Biol..

[61] Rotter A., Usadel B., Baebler Š., Stitt M., Gruden K. (2007). Adaptation of the MapMan ontology to biotic stress responses: Application in solanaceous species. Plant Methods.

[62] Borges F., Martienssen R.A. (2015). The expanding world of small RNAs in plants. Nat. Rev. Mol. Cell Biol..

[63] Van der Hoorn R.A.L., Wulff B.B.H., Rivas S., Durrant M.C., van der Ploeg A., de Wit P.J.G.M., Jones J.D.G. (2005). Structure–Function Analysis of Cf-9, a Receptor-Like Protein with Extracytoplasmic Leucine-Rich Repeats. Plant Cell.

[64] Kuang H., Wei F., Marano M.R., Wirtz U., Wang X., Liu J., Shum W.P., Zaborsky J., Tallon L.J., Rensink W. (2005). The R1 resistance gene cluster contains three groups of independently evolving, type I R1 homologues and shows substantial structural variation among haplotypes of Solanum demissum. Plant J..

[65] Fei Z., Joung J.-G., Tang X., Zheng Y., Huang M., Lee J.M., McQuinn R., Tieman D.M., Alba R., Klee H.J. (2011). Tomato Functional Genomics Database: A comprehensive resource and analysis package for tomato functional genomics. Nucleic Acids Res..

[66] Zuo J., Wang Q., Han C., Ju Z., Cao D., Zhu B., Luo Y., Gao L. (2017). SRNAome and degradome sequencing analysis reveals specific regulation of sRNA in response to chilling injury in tomato fruit. Physiol. Plant.

[67] Pan C., Ye L., Zheng Y., Wang Y., Yang D., Liu X., Chen L., Zhang Y., Fei Z., Lu G. (2017). Identification and expression profiling of microRNAs involved in the stigma exsertion under high-temperature stress in tomato. BMC Genomics.

[68] Rogers K., Chen X. (2013). Biogenesis, turnover, and mode of action of plant microRNAs. Plant Cell.

[69] Chapman E.J., Prokhnevsky A.I., Gopinath K., Dolja V.V., Carrington J.C. (2004). Viral RNA silencing suppressors inhibit the microRNA pathway at an intermediate step. Genes Dev..

[70] Lakatos L., Csorba T., Pantaleo V., Chapman E.J., Carrington J.C., Liu Y.P., Dolja V.V., Calvino L.F., López-Moya J.J., Burgyán J. (2006). Small RNA binding is a common strategy to suppress RNA silencing by several viral suppressors. EMBO J..

[71] Shiboleth Y.M., Haronsky E., Leibman D., Arazi T., Wassenegger M., Whitham S.A., Gaba V., Gal-On A. (2007). The conserved FRNK box in HC-Pro, a plant viral suppressor of gene silencing, is required for small RNA binding and mediates symptom development. J. Virol..

[72] Ye K., Malinina L., Patel D.J. (2003). Recognition of small interfering RNA by a viral suppressor of RNA silencing. Nature.

[73] Chávez Montes R.A., Rosas-Cárdenas D.F.F., De Paoli E., Accerbi M., Rymarquis L.A., Mahalingam G., Marsch-Martínez N., Meyers B.C., Green P.J., de Folter S. (2014). Sample sequencing of vascular plants demonstrates widespread conservation and divergence of microRNAs. Nat. Commun..

[74] Jeong D.-H. (2016). Functional diversity of microRNA variants in plants. J. Plant Biol..

[75] Zhang W., Gao S., Zhou X., Xia J., Chellappan P., Zhou X., Zhang X., Jin H. (2010). Multiple distinct small RNAs originate from the same microRNA precursors. Genome Biol..

[76] Du P., Wu J., Zhang J., Zhao S., Zheng H., Gao G., Wei L., Li Y. (2011). Viral infection induces expression of novel phased microRNAs from conserved cellular microRNA precursors. PLoS Pathog..

[77] Hu Q., Hollunder J., Niehl A., Kørner C.J., Gereige D., Windels D., Arnold A., Kuiper M., Vazquez F., Pooggin M. (2011). Specific Impact of Tobamovirus Infection on the Arabidopsis Small RNA Profile. PLoS ONE.

[78] Jones-Rhoades M.W., Bartel D.P., Bartel B. (2006). MicroRNAs and their regulatory roles in plants. Annu. Rev. Plant Biol..

[79] Islam W., Qasim M., Noman A., Adnan M., Tayyab M., Farooq T.H., Wei H., Wang L. (2018). Plant microRNAs: Front line players against invading pathogens. Microb. Pathog..

[80] Yang J., Zhang F., Li J., Chen J.-P., Zhang H.-M. (2016). Integrative Analysis of the microRNAome and Transcriptome Illuminates the Response of Susceptible Rice Plants to Rice Stripe Virus. PLoS ONE.

[81] Shivaprasad P.V., Chen H.-M., Patel K., Bond D.M., Santos B.A., Baulcombe D.C. (2012). A microRNA superfamily regulates nucleotide binding site–leucine-rich repeats and other mRNAs. Plant Cell.

[82] Tsushima D., Adkar-Purushothama C.R., Taneda A., Sano T. (2015). Changes in relative expression levels of viroid-specific small RNAs and microRNAs in tomato plants infected with severe and mild symptom-inducing isolates of Potato spindle tuber viroid. J. Gen. Plant Pathol..

[83] Wang W., Luan Y. (2015). The advance of tomato disease-related microRNAs. Plant Cell Rep..

[84] Chiumenti M., Catacchio C.R., Miozzi L., Pirovano W., Ventura M., Pantaleo V. (2018). A Short Indel-Lacking-Resistance Gene Triggers Silencing of the Photosynthetic Machinery Components Through TYLCSV-Associated Endogenous siRNAs in Tomato. Front. Plant Sci..

[85] Zhai J., Jeong D.-H., De Paoli E., Park S., Rosen B.D., Li Y., González A.J., Yan Z., Kitto S.L., Grusak M.A. (2011). MicroRNAs as master regulators of the plant NB-LRR defense gene family via the production of phased, trans-acting siRNAs. Genes Dev..

[86] Deng Y., Wang J., Tung J., Liu D., Zhou Y., He S., Du Y., Baker B., Li F. (2018). A role for small RNA in regulating innate immunity during plant growth. PLoS Pathog..

[87] Seo J.K., Wu J., Lii Y., Li Y., Jin H. (2013). Contribution of small RNA pathway components in plant immunity. Mol. Plant Microbe Interact..

[88] Park J.H., Shin C. (2015). The role of plant small RNAs in NB-LRR regulation. Brief. Funct. Genomics.

[89] Gonzalez V.M., Muller S., Baulcombe D., Puigdomenech P. (2015). Evolution of NBS-LRR gene copies among Dicot plants and its regulation by members of the miR482/2118 superfamily of miRNAs. Mol. Plant.

[90] Canto-Pastor A., Santos B.A., Valli A.A., Summers W., Schornack S., Baulcombe D.C. (2019). Enhanced resistance to bacterial and oomycete pathogens by short tandem target mimic RNAs in tomato. Proc. Natl. Acad. Sci. USA.

[91] Wang Z., Hardcastle T.J., Canto Pastor A., Yip W.H., Tang S., Baulcombe D.C. (2018). A novel DCL2-dependent miRNA pathway in tomato affects susceptibility to RNA viruses. Genes Dev..

[92] Bengyella L., Waikhom S.D., Allie F., Rey C. (2015). Virus tolerance and recovery from viral induced-symptoms in plants are associated with transcriptome reprograming. Plant Mol. Biol..

