# Supplementary material for: Potato Virus Y Infection Alters Small RNA Metabolism and Immune Response in Tomato
Source: Viruses. 2019 Nov 27;11(12):1100. doi: 10.3390/v11121100 (PMC6950276; doi:10.3390/v11121100)
Supplement: Supplementary file 1 [file viruses-11-01100-s001.zip › viruses-638157-suppl.pptx]

## Slide 1
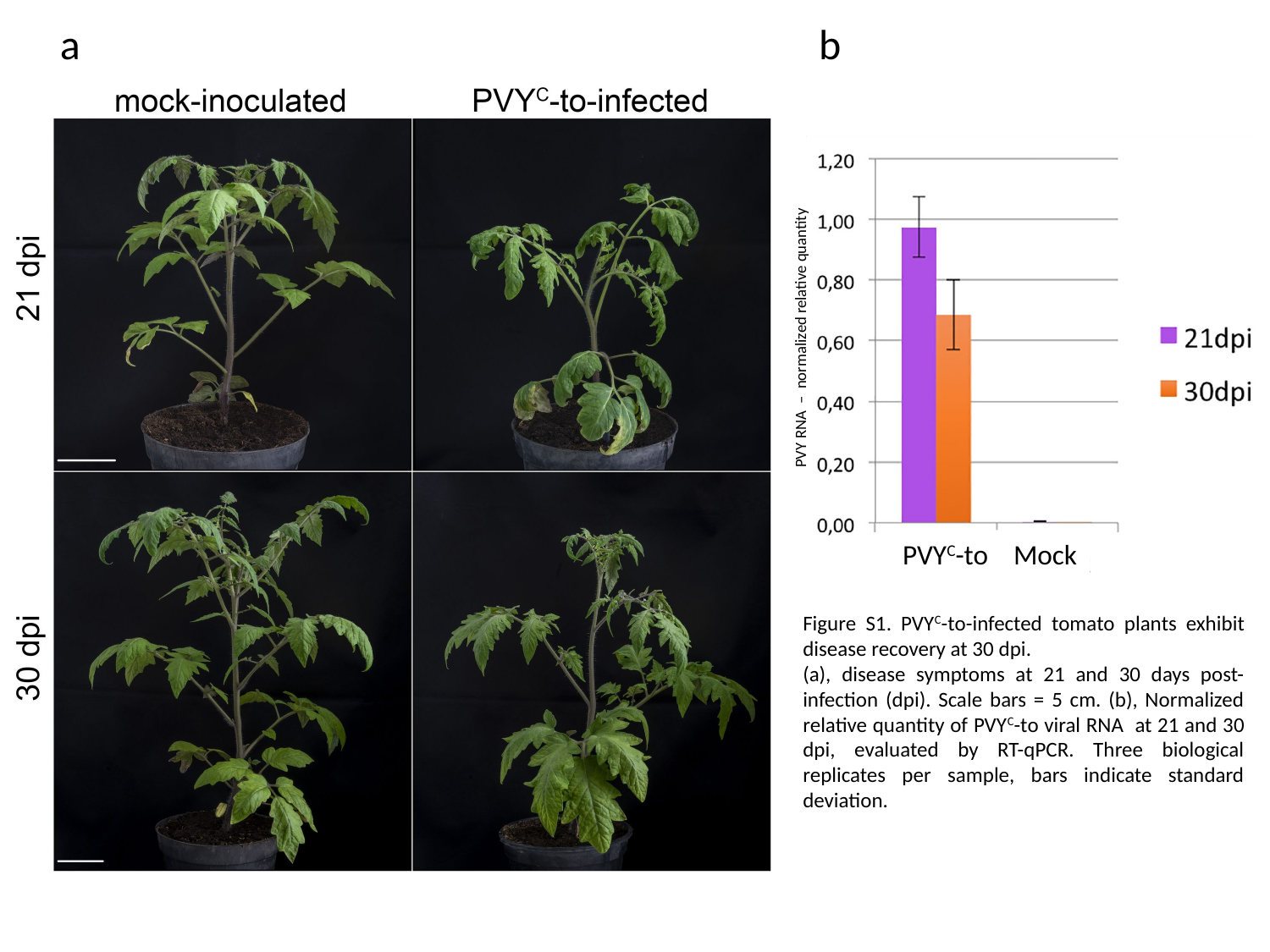

a
b
PVY RNA – normalized relative quantity
PVYC-to Mock
Figure S1. PVYC-to-infected tomato plants exhibit disease recovery at 30 dpi.
(a), disease symptoms at 21 and 30 days post-infection (dpi). Scale bars = 5 cm. (b), Normalized relative quantity of PVYC-to viral RNA at 21 and 30 dpi, evaluated by RT-qPCR. Three biological replicates per sample, bars indicate standard deviation.

## Slide 2
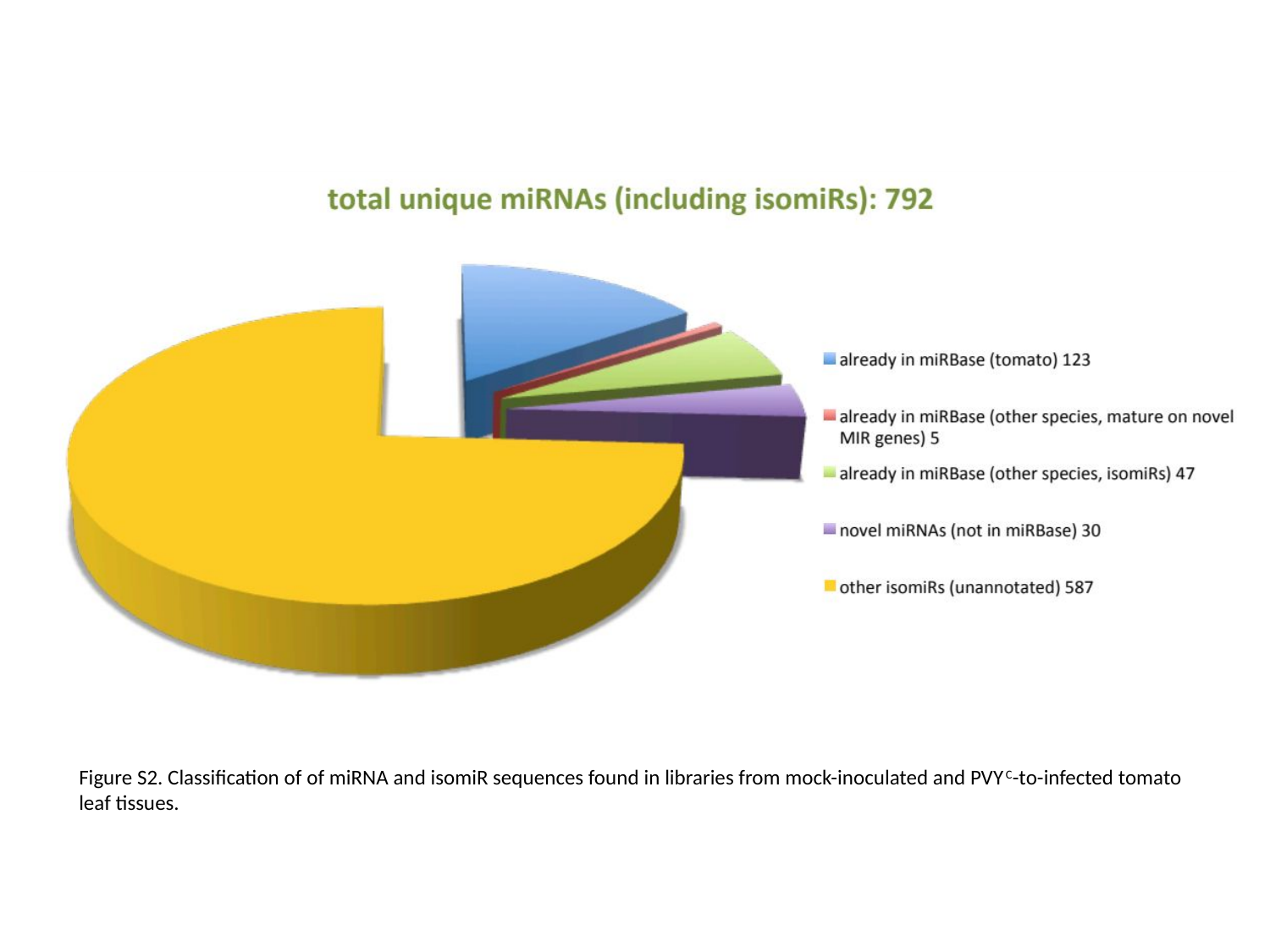

Figure S2. Classification of of miRNA and isomiR sequences found in libraries from mock-inoculated and PVYC-to-infected tomato leaf tissues.

## Slide 3
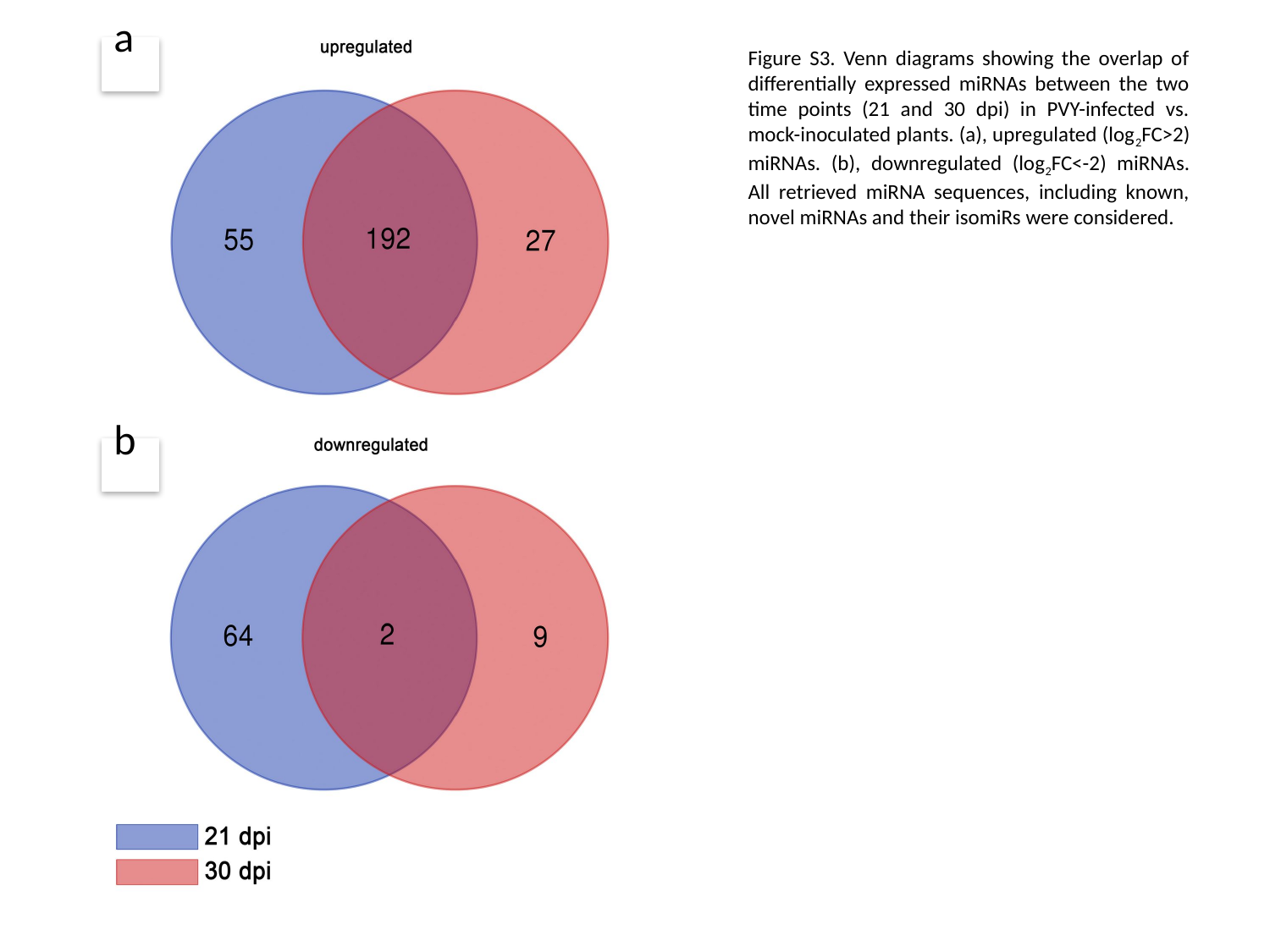

a
b
Figure S3. Venn diagrams showing the overlap of differentially expressed miRNAs between the two time points (21 and 30 dpi) in PVY-infected vs. mock-inoculated plants. (a), upregulated (log2FC>2) miRNAs. (b), downregulated (log2FC<-2) miRNAs. All retrieved miRNA sequences, including known, novel miRNAs and their isomiRs were considered.

## Slide 4
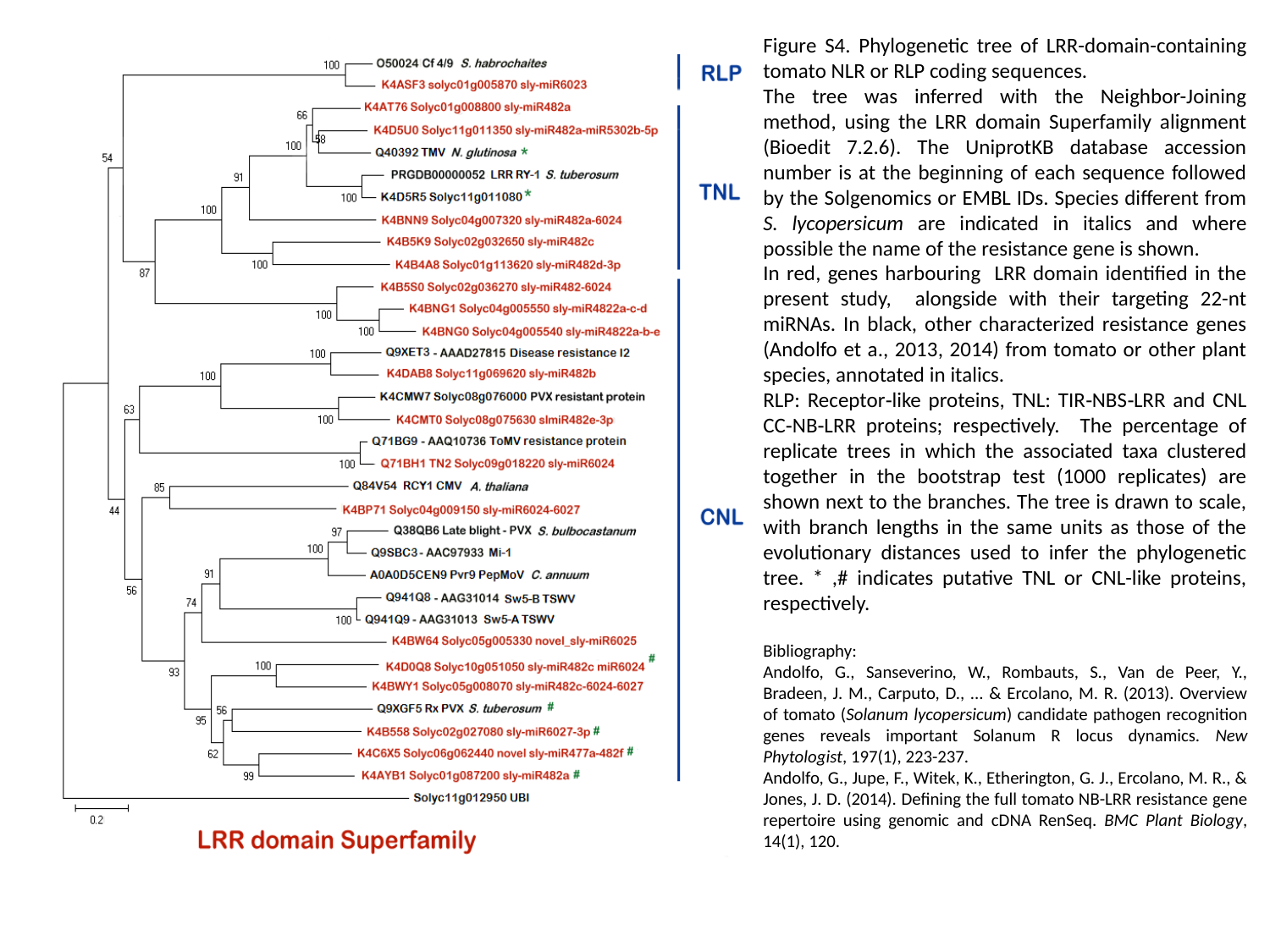

Figure S4. Phylogenetic tree of LRR-domain-containing tomato NLR or RLP coding sequences.
The tree was inferred with the Neighbor-Joining method, using the LRR domain Superfamily alignment (Bioedit 7.2.6). The UniprotKB database accession number is at the beginning of each sequence followed by the Solgenomics or EMBL IDs. Species different from S. lycopersicum are indicated in italics and where possible the name of the resistance gene is shown.
In red, genes harbouring LRR domain identified in the present study, alongside with their targeting 22-nt miRNAs. In black, other characterized resistance genes (Andolfo et a., 2013, 2014) from tomato or other plant species, annotated in italics.
RLP: Receptor‐like proteins, TNL: TIR‐NBS‐LRR and CNL CC‐NB‐LRR proteins; respectively. The percentage of replicate trees in which the associated taxa clustered together in the bootstrap test (1000 replicates) are shown next to the branches. The tree is drawn to scale, with branch lengths in the same units as those of the evolutionary distances used to infer the phylogenetic tree. * ,# indicates putative TNL or CNL-like proteins, respectively.
Bibliography:
Andolfo, G., Sanseverino, W., Rombauts, S., Van de Peer, Y., Bradeen, J. M., Carputo, D., ... & Ercolano, M. R. (2013). Overview of tomato (Solanum lycopersicum) candidate pathogen recognition genes reveals important Solanum R locus dynamics. New Phytologist, 197(1), 223-237.
Andolfo, G., Jupe, F., Witek, K., Etherington, G. J., Ercolano, M. R., & Jones, J. D. (2014). Defining the full tomato NB-LRR resistance gene repertoire using genomic and cDNA RenSeq. BMC Plant Biology, 14(1), 120.

## Slide 5
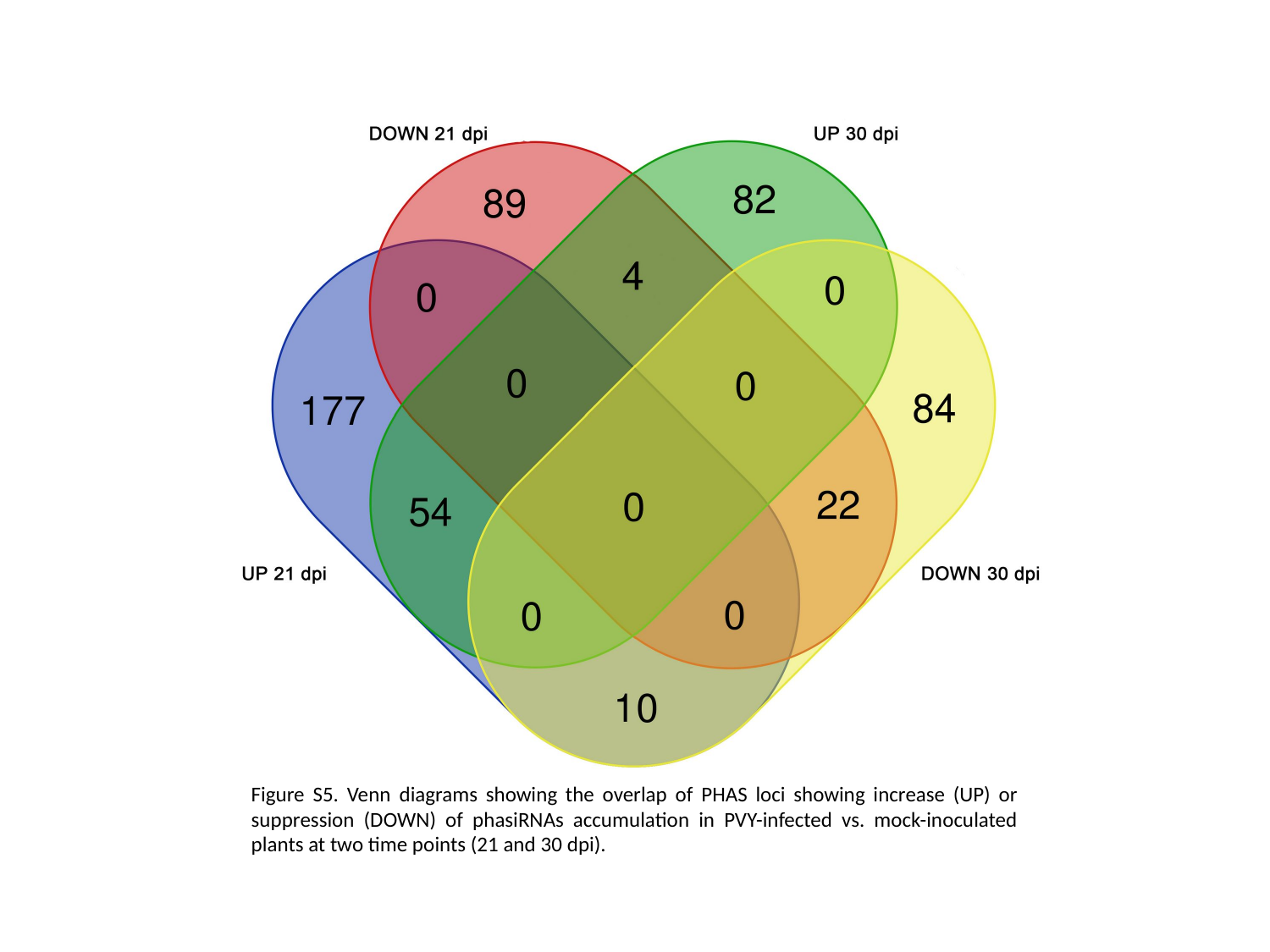

Figure S5. Venn diagrams showing the overlap of PHAS loci showing increase (UP) or suppression (DOWN) of phasiRNAs accumulation in PVY-infected vs. mock-inoculated plants at two time points (21 and 30 dpi).

## Slide 6
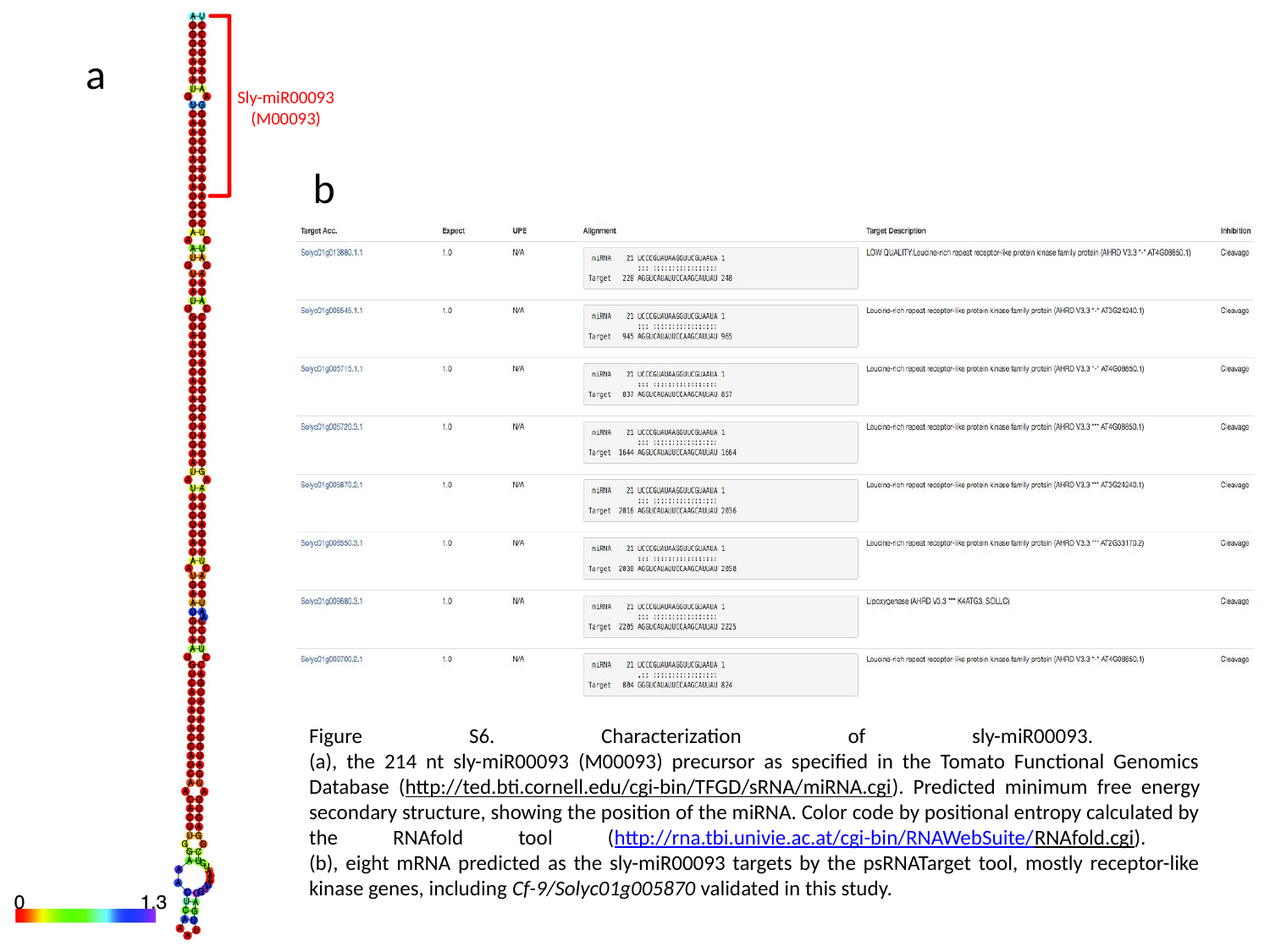

a
Sly-miR00093
(M00093)
b
Figure S6. Characterization of sly-miR00093.
(a), the 214 nt sly-miR00093 (M00093) precursor as specified in the Tomato Functional Genomics Database (http://ted.bti.cornell.edu/cgi-bin/TFGD/sRNA/miRNA.cgi). Predicted minimum free energy secondary structure, showing the position of the miRNA. Color code by positional entropy calculated by the RNAfold tool (http://rna.tbi.univie.ac.at/cgi-bin/RNAWebSuite/RNAfold.cgi).
(b), eight mRNA predicted as the sly-miR00093 targets by the psRNATarget tool, mostly receptor-like kinase genes, including Cf-9/Solyc01g005870 validated in this study.
